# Supplementary material for: OVOL2 sustains postnatal thymic epithelial cell identity
Source: Nat Commun. 2023 Nov 27;14:7786. doi: 10.1038/s41467-023-43456-z (PMC10682436; doi:10.1038/s41467-023-43456-z)
Supplement: Supplementary file 1 — Supplementary Information [file 41467_2023_43456_MOESM1_ESM.pdf]

Supplementary Information

OVOL2 sustains postnatal thymic epithelial cell identity

Xue Zhong,[1†] Nagesh Peddada,[1†] Jianhui Wang,[1] James J. Moresco,[1] Xiaowei Zhan,[1,2] John M. Shelton,[3] Jeffrey A. SoRelle,[4,5] Katie Keller,[1] Danielle Renee. Lazaro,[1] Eva Marie Y. Moresco,[1] Jin Huk Choi[1]* and Bruce Beutler[1]*

[1]Center for the Genetics of Host Defense, University of Texas Southwestern Medical Center, Dallas, Texas, 75390-8505, USA.

[2]Department of Population and Data Sciences, Quantitative Biomedical Research Center, University of Texas Southwestern Medical Center, Dallas, Texas, 75390-8821, USA.

[3]Intermal Medicine-Histopathology Core, University of Texas Southwestern Medical Center, Dallas, Texas, 75390-8573, USA.

[4]Department of Pathology, University of Texas Southwestern Medical Center, Dallas, Texas, 75390-9072, USA.

[5]Department of Pediatrics, University of Texas Southwestern Medical Center, Dallas, Texas, 75390-9063, USA.

*Corresponding authors. Email: Jin.Choi@UTSouthwestern.edu or Bruce.Beutler@UTSouthwestern.edu.

[†]These authors contributed equally to this work.

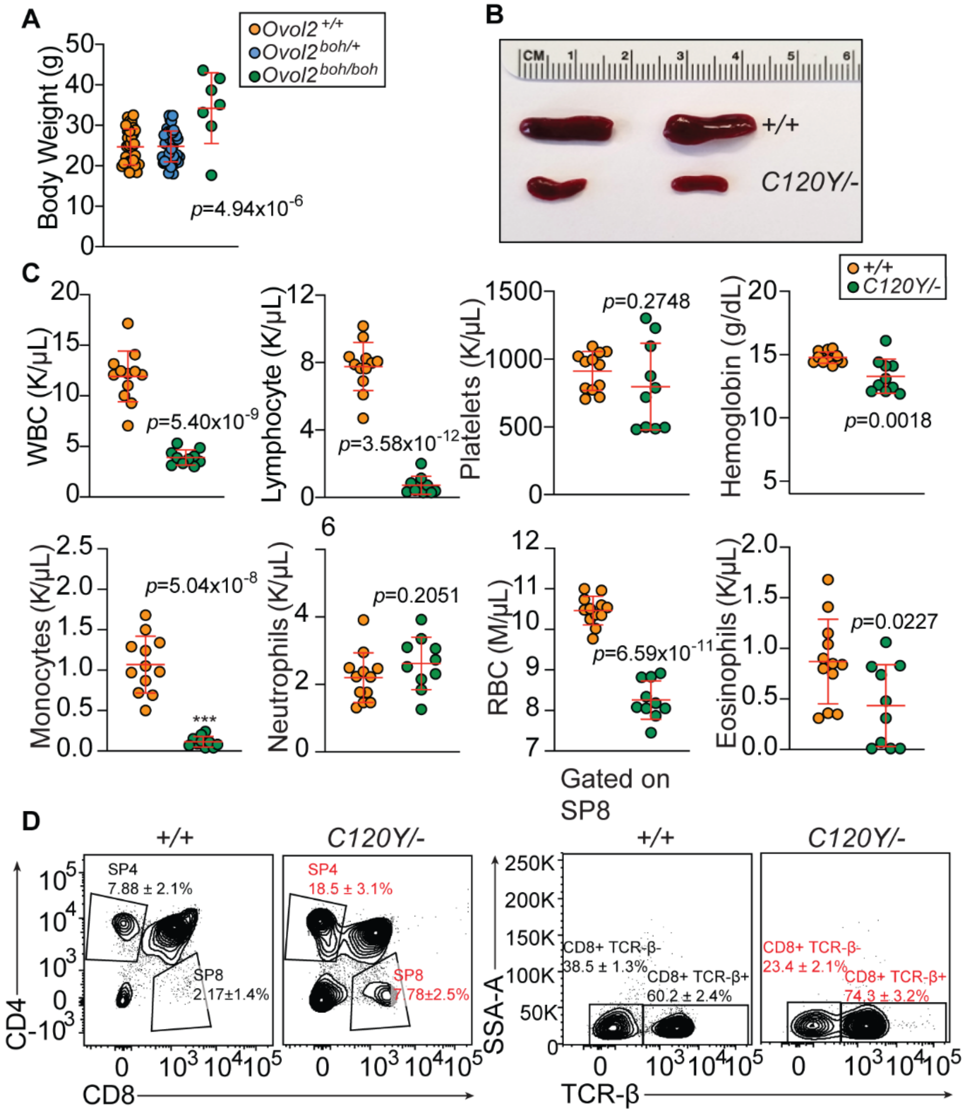

Supplementary Figure 1. Elevated body weight, hypoplastic spleens, lymphopenia, monocytopenia, anemia, and thymocyte developmental defects caused by an *Ovol2* mutation.

(**A**) Body weights of third generation (G3) descendants of a single ENU-mutagenized male mouse with the indicated genotypes for *Ovol2* (*n* =7 *Ovol2*$^{boh/boh}$, 50 *Ovol2*$^{boh/+}$, 35 WT littermates). (**B**) Representative photographs of spleens isolated from 12-wk-old *Ovol2*$^{C120Y/-}$ and WT littermates. (**C**) Complete blood count testing. White blood cells (WBCs), lymphocytes, monocytes, neutrophils, red blood cells (RBCs), eosinophils, platelets, and hemoglobin in 12-wk-old *Ovol2*$^{C120Y/-}$ and WT littermates (*n* =10 *C120Y/-*, 12 WT littermates). (D) Representative flow cytometry plots of thymocyte populations in 12-wk-old *Ovol2*$^{C120Y/-}$ mice and WT littermates. Numbers adjacent to outlined regions indicate percent cells in each (mean ± SD) (*n* =3 mice/genotype). Data are representative of one experiment (A) or two independent experiments (C-D). Data points represent individual mice and means ± SD are indicated. *P* values were determined by one-way analysis of variance (ANOVA) with Dunnett's multiple comparisons (A) or two-tailed Student's *t* test (C). +/+ indicates WT and *C120Y/-* indicates the *Ovol2*$^{C120Y/-}$ genotype. SP, single positive.

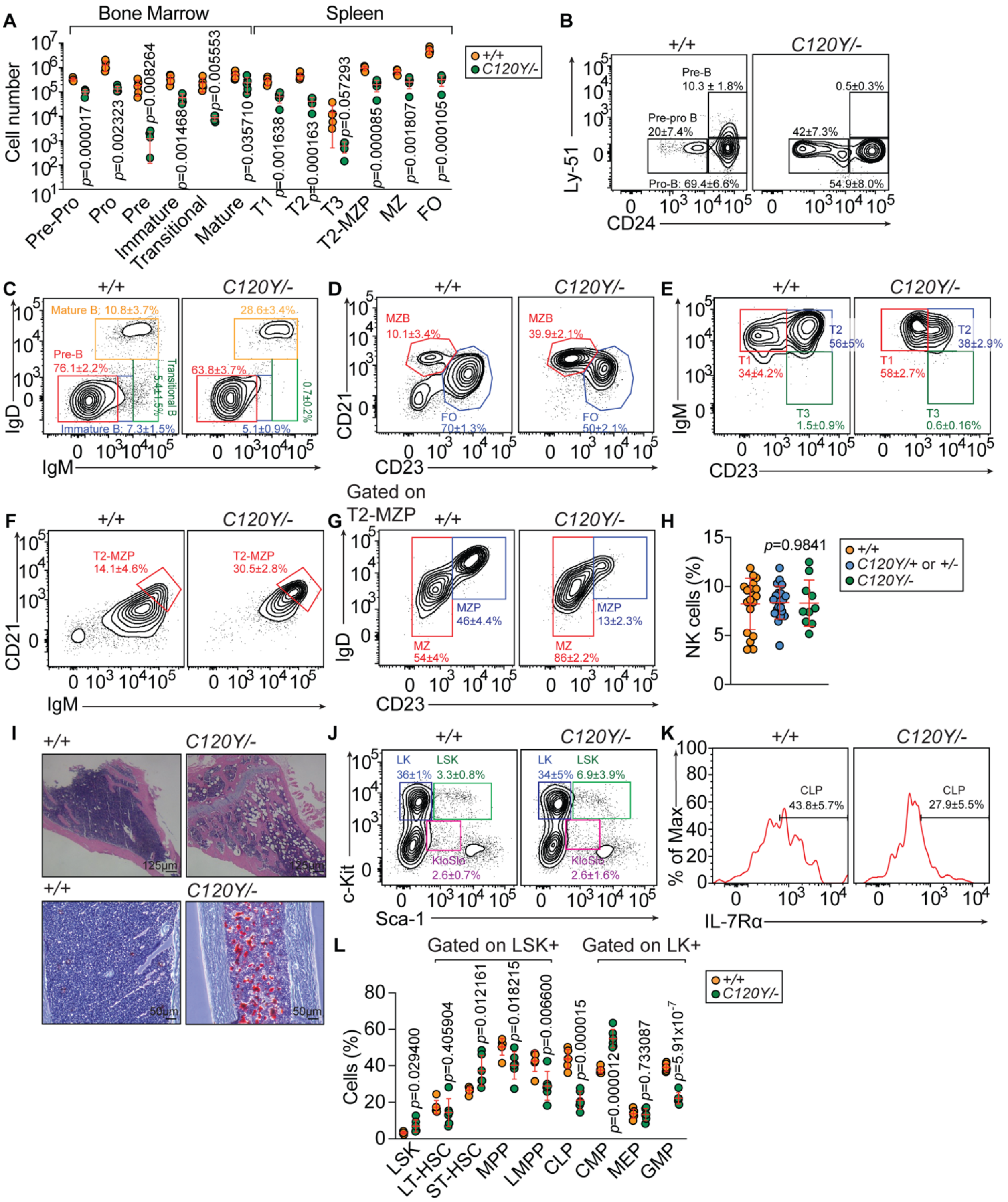

Supplementary Figure 2. *Ovol2* mutation is responsible for deficiencies of hematopoietic stem cells (HSC), B cell development, and NK cell function.

(**A**) Quantitative analysis of B cell subpopulations in the bone marrow (BM) and spleen of 12-wk-old *Ovol2*$^{C120Y/-}$ and WT littermates (*n* =5 mice/genotype). (**B-G**) Representative flow cytometry plots showing B cell development in the BM (B and C) and spleen (D-G) from 12-wk-old

Ovol2$^{C120Y/-}$ and WT littermates (*n* =5 mice/genotype). Each B cell subset was gated as follows: pre-pro-B: $B220^{low}Ly51^{-}CD24^{low}$, pro-B: $B220^{low}Ly51^{-}CD24^{+}$, pre-B: $B220^{low}Ly51^{+}CD24^{+}$, immature B: $B220^{+}IgM^{+}IgD^{-}$, transitional B: $B220^{+}IgM^{high}IgD^{low}$, mature recirculating B: $B220^{+}IgD^{+}IgM^{+}$, T1: $B220^{+}CD93^{+}IgM^{high}CD23^{-}$, T2: $B220^{+}CD93^{+}IgM^{high}CD23^{+}$, T3: $B220^{+}CD93^{+}IgM^{low}CD23^{+}$, follicular B (FOB): $B220^{+}CD93^{-}IgM^{+}CD21^{+}CD23^{high}$, marginal zone precursor (MZP): $B220^{+}CD93^{-}IgM^{+}CD21^{+}CD2a3^{high}$, and marginal zone B (MZB): $B220^{+}CD93^{-}IgM^{+}CD21^{+}CD23^{low}$. Numbers adjacent or inside outlined regions represent percent cells in each (mean ± SD) (*n* =5 mice/genotype). (**H**) Frequency of NK cells in the peripheral blood (*n* =10 *C120Y/-*, 22 *C120Y/+* or +/-, 19 WT littermates). (**I**) Representative hematoxylin and eosin staining (upper) and Oil red O staining (lower) of WT and *Ovol2*$^{C120Y/-}$ littermate bone sections at 12 wk of age. (**J** and **K)**, Representative flow cytometry plots of Lineage-marker negative (Lin^{-}) populations (J) and common lymphoid progenitor (CLP) (K) from *Ovol2*$^{C120Y/-}$ or WT littermates. Numbers adjacent to outlined regions represent percent cells in each (mean ± SD) (*n* =5 mice/genotype). (**L**) Flow cytometry analysis of HSC and progenitor populations in the BM of 12-wk-old *Ovol2*$^{C120Y/-}$ and WT littermates (*n* =6 mice/genotype). Data are representative of one experiment (H) or two independent experiments (A-G and I-L). Data points represent individual mice and means ± SD are indicated. *P* values were determined by one-way analysis of variance (ANOVA) with Dunnett's multiple comparisons (H) or two-tailed Student's *t* test (A and L). +/+, *C120Y/+*, and *C120Y/-* indicate WT, *Ovol2*$^{C120Y/+}$, and *Ovol2*$^{C120Y/-}$ genotypes, respectively. LSK (c-Kit+ Sca-1+); LK (c-Kit+ Sca-1–); and $K_{lo}S_{lo}$ (c-Kit_{low} Sca-1_{low}); long-term hematopoietic stem cells (LT-HSC); short-term hematopoietic stem cells (ST-HSC); multipotent progenitors (MPP); lymphoid-primed multipotent progenitors (LMPP); megakaryocyte–erythroid progenitors (MEP); common myeloid progenitors (CMP); and granulocyte-macrophage progenitors (GMP).

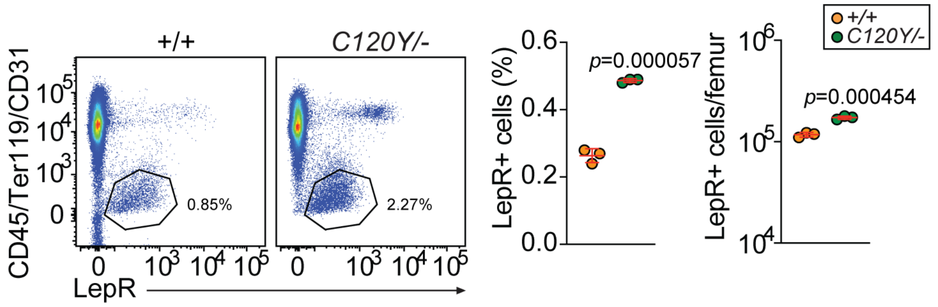

Supplementary Figure 3. Elevated LepR+ cells in *Ovol2*$^{C120Y/-}$ bone marrow.

Representative flow cytometry plots (left) and quantitative analysis (right) of LepR+ cells in bone marrow from 12-wk-old *Ovol2*$^{C120Y/-}$ mice and WT littermates. Numbers adjacent to outlined regions indicate representative percentage in each population (n =3 mice/genotype). Data are representative of two independent experiments. Data points represent individual mice and means ± SD are indicated. P values were determined by two-tailed Student's t test. +/+ indicates WT and *C120Y/-* indicates the *Ovol2*$^{C120Y/-}$ genotype.

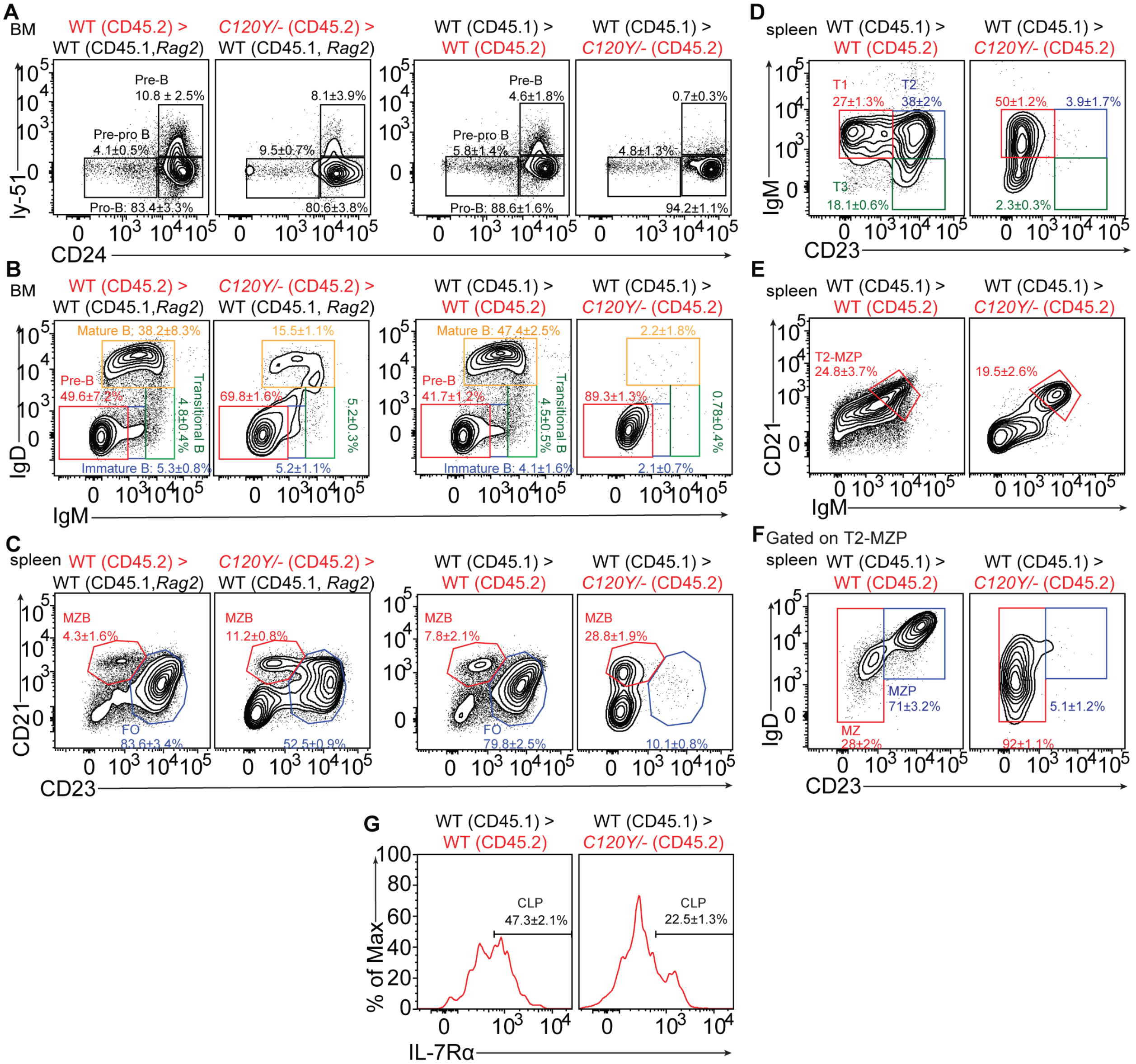

Supplementary Figure 4. Hematopoietic intrinsic and extrinsic effects of *Ovol2* mutation on B cell development.

(**A-F**) Representative flow cytometry plots showing repopulation of B cells in bone marrow (A and B) and spleen (C-F) 12 wk after reconstitution of lethally irradiated recipients indicated in figure. (**A-C**, left) WT BM (CD45.2) or *Ovol2*$^{C120Y/-}$ BM (CD45.2) was injected into lethally irradiated *Rag2*$^{-/-}$ (CD45.1) recipients. (**A-C**, right) and (**D-F**) WT BM (CD45.1) was injected into lethally irradiated WT (CD45.2) or *Ovol2*$^{C120Y/-}$ (CD45.2) recipients. Numbers adjacent to outlined regions indicate percent cells in each (mean ± SD) (*n* =3 recipients per group). (**G**) Representative flow cytometry plots showing repopulation of common lymphoid progenitors (CLP) 12 wk after BMT from congenic WT mice (C57BL/6J; CD45.1) to lethally irradiated WT (CD45.2) or *Ovol2*$^{C120Y/-}$ (CD45.2) recipients. Numbers adjacent to bracketed regions indicate percent cells in each (mean ± SD) (*n* =5 recipients per group). Data are representative of two independent experiments. *C120Y/-* indicates the *Ovol2*$^{C120Y/-}$ genotype.

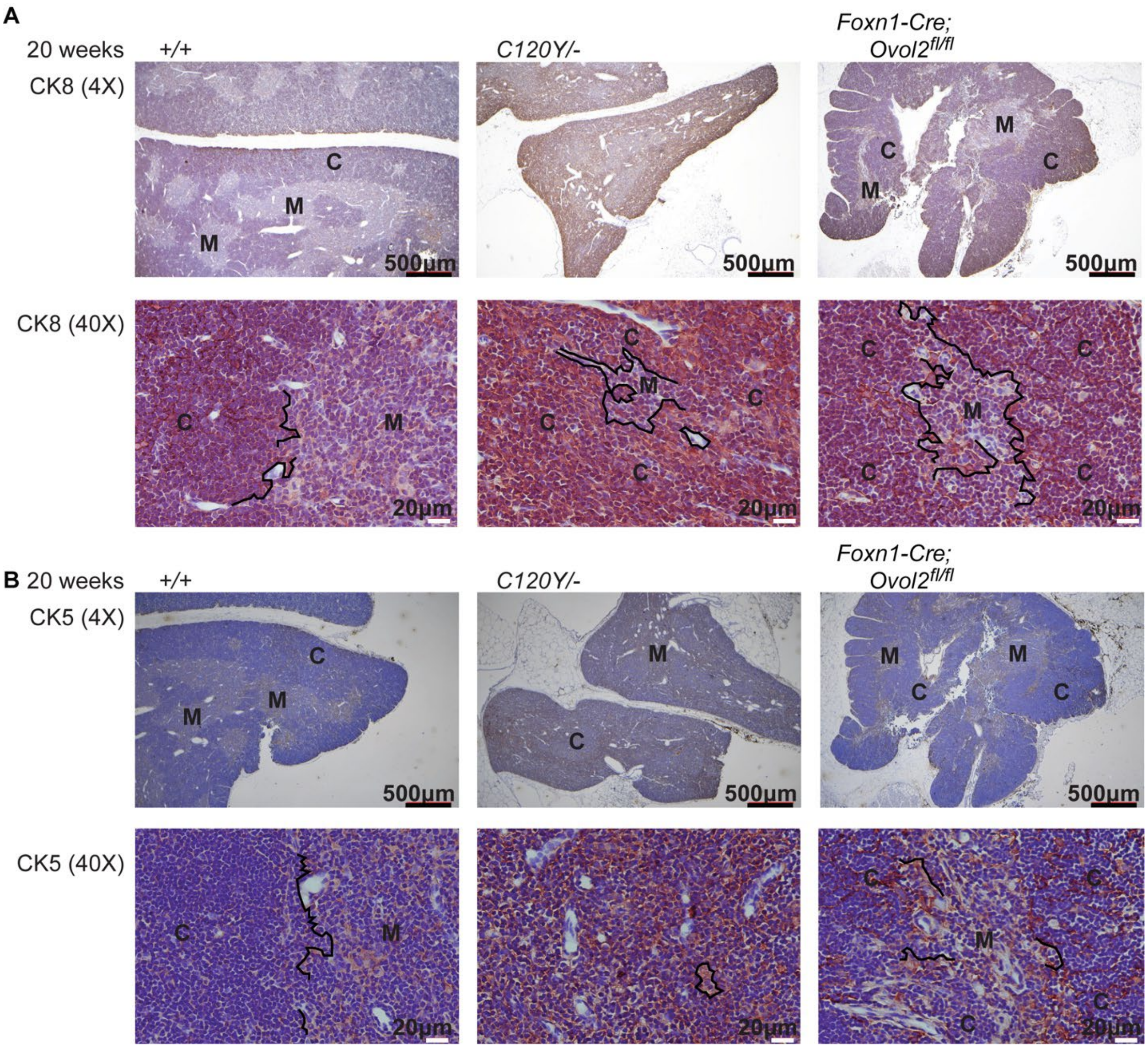

Supplementary Figure 5. Immunohistochemical analysis of cytokeratins in *Ovol2*$^{C120Y/-}$ and *Foxn1-cre*;*Ovol2*$^{fl/fl}$ thymi.

(**A**-**B**) Cytokeratin staining of thymus sections from 20-wk-old *Ovol2*$^{C120Y/-}$, *Foxn1-cre*;*Ovol2*$^{fl/fl}$, and WT mice. (A) Cytokeratin (CK) 8 labels cortical epithelium. Whereas CK8 clearly labels the cortical regions and is absent from the medullary regions of WT thymus, the ubiquitous CK8 staining in the *Ovol2*$^{C120Y/-}$ and *Foxn1-cre;Ovol2*$^{fl/fl}$ thymi suggests that most of the medullary epithelium has been lost. (B) CK5 label medullary epithelium. Whereas CK5 clearly label the medullary regions and are absent from the cortical regions of WT thymus, the weak and diffuse ubiquitous staining in *Ovol2*$^{C120Y/-}$ and *Foxn1-cre;Ovol2*$^{fl/fl}$ thymi suggests low levels of these epitopes. In the mutant thymi, the medullary regions are smaller and the cortical regions are larger than in WT thymi. For WT samples, sections of a portion of the thymus are shown; for mutant samples, sections of the whole thymus are shown. C, cortical region; M, medullary region. Boundaries of C and M are indicated where staining could be distinguished. +/+, WT; *C120Y/-*, *Ovol2*$^{C120Y/-}$.

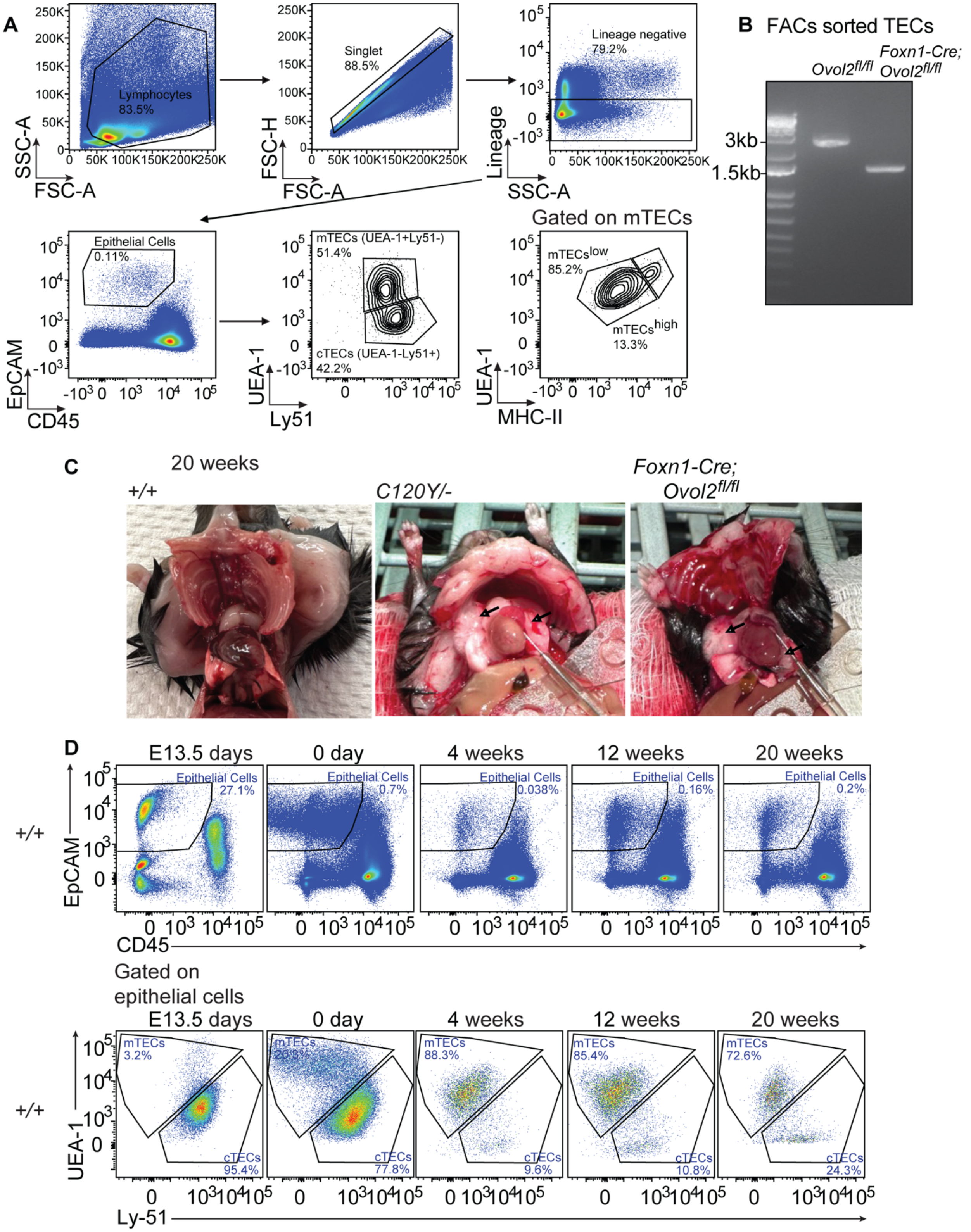

Supplementary Figure 6. Gating strategy for TECs, detection of adipose tissue in OVOL2 deficient mice, and mTEC vs. cTEC frequencies at several ages during embryogenesis and postnatally.

(**A**) Flow cytometry gating strategy to detect mTECs and cTECs in the thymus. (**B**) PCR confirmation of deletion of exon 3 cassette containing 1177 bp of *Ovol2* genomic DNA, of which 190 bp are coding sequence, in FACS sorted *Foxn1-Cre*;*Ovol2*$^{fl/fl}$ TECs. (**C**) Representative photographs of thoracic cavity from 20-wk-old *Ovol2*$^{C120Y/-}$, *Foxn1-Cre*, *Ovol2*$^{fl/fl}$ and age matched WT mice. Note the presence of adipose tissue throughout the thoracic cavity of *Ovol2*$^{C120Y/-}$ mice but only surrounding the thymus in *Foxn1-Cre*, *Ovol2*$^{fl/fl}$ mice (black arrows). (**D**) Representative flow cytometry plots of TECs from WT mice at the indicated ages. +/+, WT; and *C120Y/-*, *Ovol2*$^{C120Y/-}$.

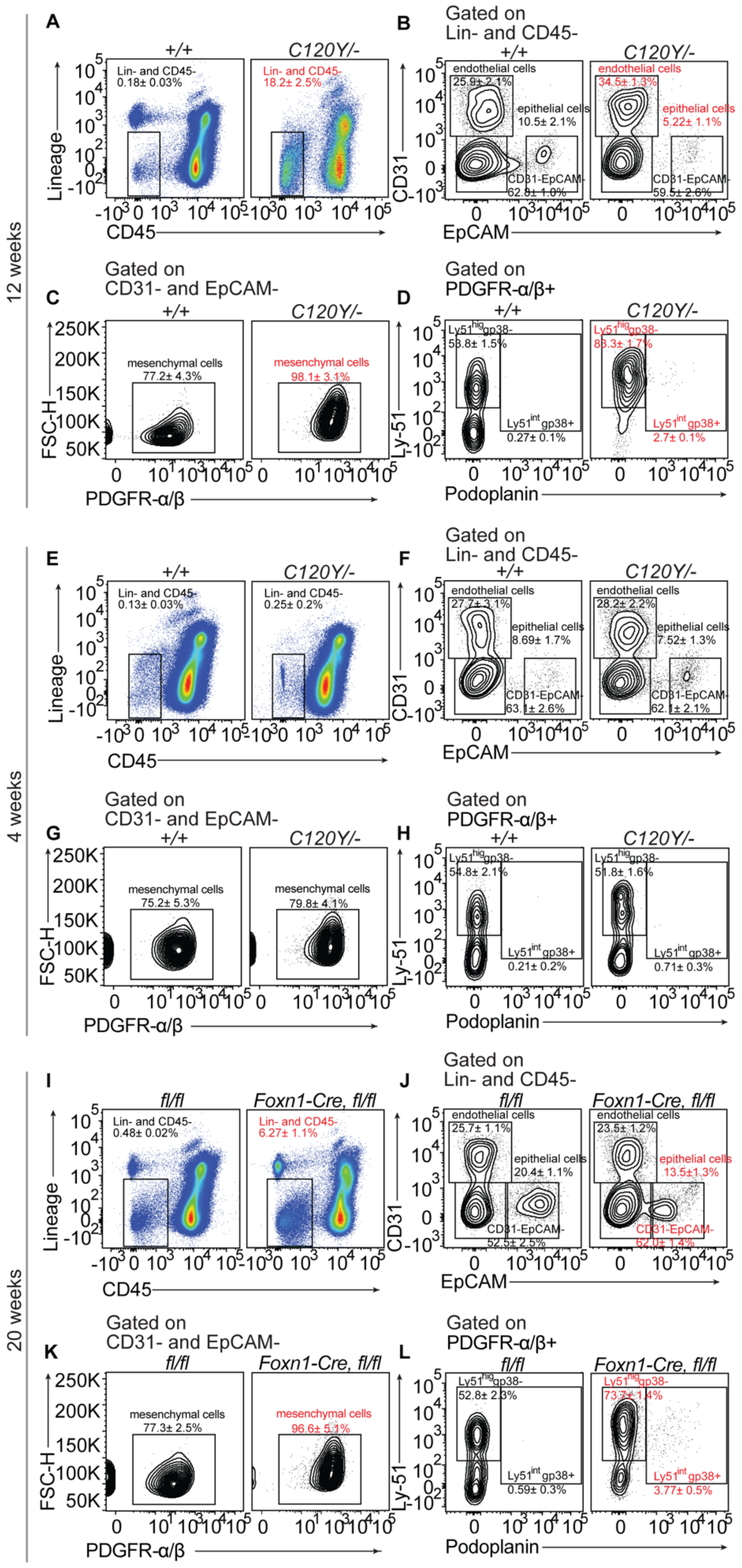

12 weeks
A
+/+
C120Y/-
Lin- and CD45-
0.18± 0.03%
Lin- and CD45-
18.2± 2.5%
Lineage
CD45
B
Gated on
Lin- and CD45-
endothelial cells
25.9± 2.1%
epithelial cells
10.5± 2.1%
CD31-EpCAM-
62.8± 1.0%
endothelial cells
34.5± 1.3%
epithelial cells
5.22± 1.1%
CD31-EpCAM-
59.5± 2.6%
CD31
EpCAM
C
Gated on
CD31- and EpCAM-
mesenchymal cells
77.2± 4.3%
mesenchymal cells
98.1± 3.1%
FSC-H
PDGFR-α/β
D
Gated on
PDGFR-α/β+
Ly51^hig gp38-
53.8± 1.5%
Ly51^int gp38+
0.27± 0.1%
Ly51^hig gp38-
83.3± 1.7%
Ly51^int gp38+
2.7± 0.1%
Ly-51
Podoplanin
4 weeks
E
Lin- and CD45-
0.13± 0.03%
Lin- and CD45-
0.25± 0.2%
F
endothelial cells
27.7± 3.1%
epithelial cells
8.69± 1.7%
CD31-EpCAM-
63.1± 2.6%
endothelial cells
28.2± 2.2%
epithelial cells
7.52± 1.3%
CD31-EpCAM-
62.1± 2.1%
G
mesenchymal cells
75.2± 5.3%
mesenchymal cells
79.8± 4.1%
H
Ly51^hig gp38-
54.8± 2.1%
Ly51^int gp38+
0.21± 0.2%
Ly51^hig gp38-
51.8± 1.6%
Ly51^int gp38+
0.71± 0.3%
20 weeks
I
fl/fl
Foxn1-Cre, fl/fl
Lin- and CD45-
0.48± 0.02%
Lin- and CD45-
6.27± 1.1%
J
endothelial cells
25.7± 1.1%
epithelial cells
20.4± 1.1%
CD31-EpCAM-
52.5± 2.5%
endothelial cells
23.5± 1.2%
epithelial cells
13.5±1.3%
CD31-EpCAM-
62.0± 1.4%
K
mesenchymal cells
77.3± 2.5%
mesenchymal cells
96.6± 5.1%
L
Ly51^hig gp38-
52.8± 2.3%
Ly51^int gp38+
0.59± 0.3%
Ly51^hig gp38-
73.7± 1.4%
Ly51^int gp38+
3.77± 0.5%

Supplementary Figure 7. Frequencies of endothelial and mesenchymal cells in thymi from *Ovol2*$^{C120Y/-}$ and *Foxn1-Cre*;*Ovol2*$^{fl/fl}$ mice.

(**A**-**L**) Representative flow cytometry plots of thymic stromal cells and the indicated subpopulations from 12-wk-old (A-D) and 4-wk-old (E-H) *Ovol2*$^{C120Y/-}$ mice and WT littermates, or from 20-wk-old *Foxn1-Cre*;*Ovol2*$^{fl/fl}$ mice and *Ovol2*$^{fl/fl}$ littermates (I-L). Each subset was gated as follows: endothelial cells: EpCAM^{-}CD31^{+}, epithelial cells: EpCAM^{+}CD31^{-}, mesenchymal cells: EpCAM^{-}CD31^{-}PDGFR-α/β^{+}, fibroblasts: EpCAM^{-}CD31^{-}PDGFR-α/β^{+}Ly51^{int}gp38^{+}. Numbers adjacent or inside outlined regions represent percent cells in each (mean ± SD) (*n* =4 *C120Y/-*, 3 WT littermates in A-D; 3 mice/genotype in E-L). Data are representative of two independent experiments. +/+, WT; *C120Y/-*, *Ovol2*$^{C120Y/-}$; and *fl/fl*, *Ovol2*$^{fl/fl}$.

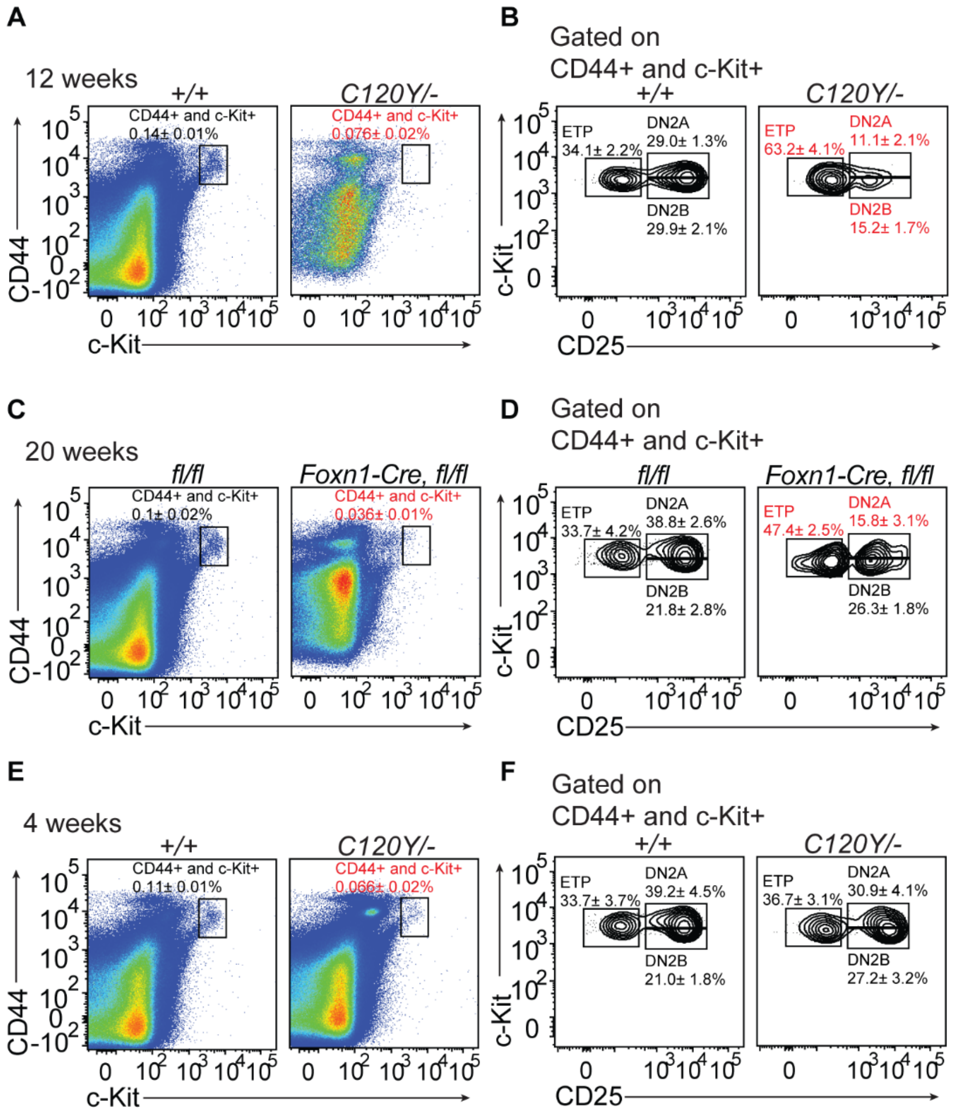

Supplementary Figure 8. Deficiency of early T-cell progenitors (ETP) in *Ovol2*$^{C120Y/-}$ and *Foxn1-Cre*;*Ovol2*$^{fl/fl}$ thymi.

(**A-F**) Representative flow cytometry plots of ETP from 12-wk-old (A-B) and 4-wk-old *Ovol2*$^{C120Y/-}$ mice (E-F) and WT littermates, or 20-wk-old *Foxn1-Cre*;*Ovol2*$^{fl/fl}$ mice and *Ovol2*$^{fl/fl}$ littermates (C-D). ETP were gated as CD44^{+}c-Kit^{+}CD25^{low}. Numbers adjacent or inside outlined regions represent percent cells in each (mean ± SD) (*n* =3 mice/genotype in A-D; 4 *C120Y/-*, 3 WT littermates in E-F). Data are representative of two independent experiments. ETP, early T-cell progenitors; DN, double negative. +/+, WT; *C120Y/-*, *Ovol2*$^{C120Y/-}$; and *fl/fl*, *Ovol2*$^{fl/fl}$.

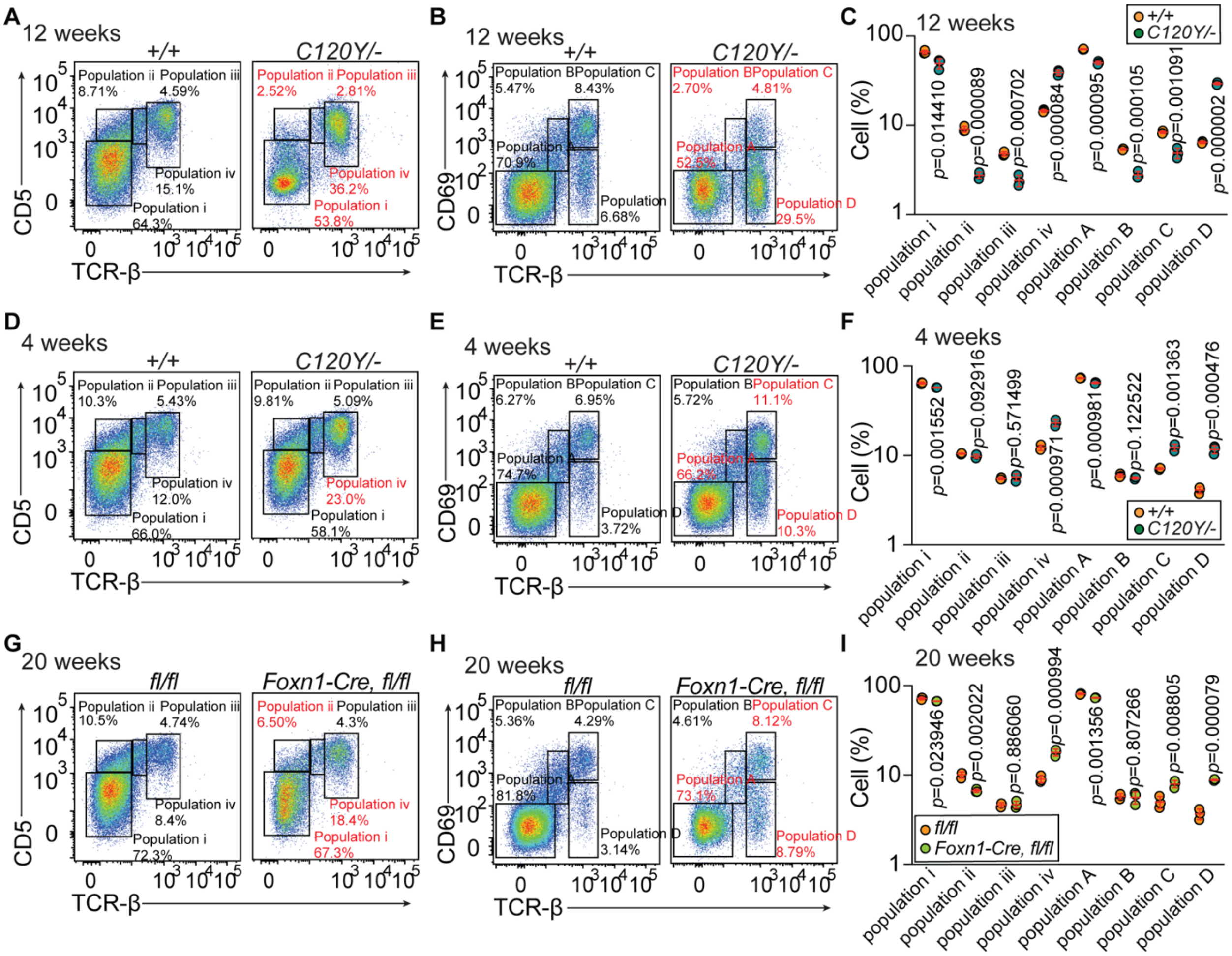

Supplementary Figure 9. Impairment of T cell positive selection in *Ovol2*$^{C120Y/-}$ and *Foxn1-Cre*;*Ovol2*$^{fl/fl}$ thymi.

(**A-I**) Representative flow cytometry plots (A-B, D-E, and G-H) and quantitative analysis (C, F, and I) of thymocytes at stages of positive selection defined by CD5 (A, D, and G) or CD69 (B, E, and H) and TCRβ cell surface expression. 12-wk-old (A-C), or 4-wk-old *Ovol2*$^{C120Y/-}$ mice (D-F) and WT littermates, or 20-wk-old *Foxn1-Cre*;*Ovol2*$^{fl/fl}$ mice and *Ovol2*$^{fl/fl}$ littermates (G-I). (**A, D, and G**) Population i (TCRβ^{lo}CD5^{lo}) represents pre-selection DP thymocytes. Population ii (TCRβ^{lo}CD5^{int}) represents cells initiating positive selection. Population iii (TCRβ^{int}CD5^{hi}) represents thymocytes in the process of positive selection. Population iv (TCRβ^{hi}CD5^{hi}) consists primarily of post-positive selection SP thymocytes. Compared to WT or *Ovol2*$^{fl/fl}$ littermates, 12-wk-old *Ovol2*$^{C120Y/-}$ mice and 20-wk-old *Foxn1-Cre*;*Ovol2*$^{fl/fl}$ mice showed decreased proportions of T cells entering positive selection (population ii). However, post-positive selection SP thymocytes (population iv) were significantly increased in frequency in 12-wk-old *Ovol2*$^{C120Y/-}$ mice and 20-wk-old *Foxn1-Cre*;*Ovol2*$^{fl/fl}$ mice. 4-wk-old *Ovol2*$^{C120Y/-}$ mice showed normal frequencies of populations ii and iii, but an increased frequency of population iv. (**B, E, and H**) Population A (TCRβ^{lo} CD69^{-}) represents pre-selection DP thymocytes. Population B (TCRβ^{int} CD69^{+}) represents a transitional population directly after TCR engagement. Population C (TCRβ^{hi} CD69^{+}) represents immediately post-positive selection cells; population D (TCRβ^{hi} CD69^{-}) represents a more mature post-positive selection population of cells. Compared

to WT or *Ovol2*$^{fl/fl}$ littermates, 12-wk-old and 4-wk-old *Ovol2*$^{C120Y/-}$ mice and 20-wk-old *Foxn1-Cre*;*Ovol2*$^{fl/fl}$ mice showed significantly increased post-positive selection cells (population D). Population B was decreased in 12-wk-old *Ovol2*$^{C120Y/-}$ mice but not in 20-wk-old *Foxn1-Cre*;*Ovol2*$^{fl/fl}$ mice. Numbers adjacent or inside outlined regions indicate representative percentage in each population (*n* =3 mice/genotype). Data are representative of two independent experiments. Data points represent individual mice and means ± SD are indicated. *P* values were determined by two-tailed Student's *t* test (C, F, and I). +/+, WT; *C120Y/-*, *Ovol2*$^{C120Y/-}$; and *fl/fl*, *Ovol2*$^{fl/fl}$.

Supplementary Fig. 10

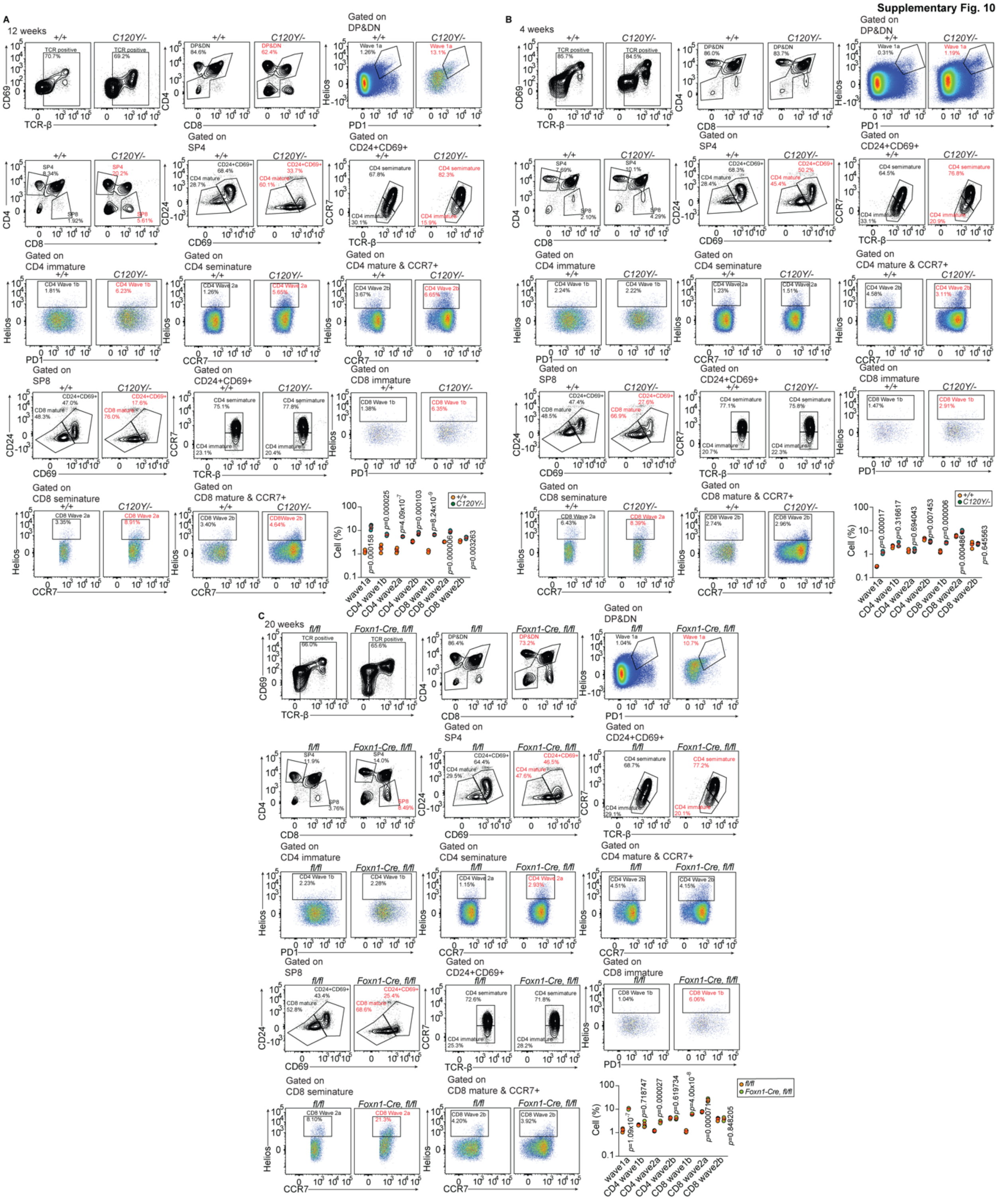

Supplementary Figure 10. Altered T cell negative selection in *Ovol2*$^{C120Y/-}$ and *Foxn1-Cre*;*Ovol2*$^{fl/fl}$ thymi.

(**A-C**) Representative flow cytometry plots and quantitative analysis of thymocytes at stages of negative selection defined as in ref. 1. 12-wk-old (A), or 4-wk-old *Ovol2*$^{C120Y/-}$ mice (B) and WT littermates, or 20-wk-old *Foxn1-Cre*;*Ovol2*$^{fl/fl}$ mice and *Ovol2*$^{fl/fl}$ littermates (C). Numbers adjacent

or inside outlined regions indicate representative percentage in each population (*n* =4 mice/genotype). Data are representative of two independent experiments. Data points represent individual mice and means ± SD are indicated. *P* values were determined by two-tailed Student's *t* test. +/+, WT; *C120Y/-*, $Ovol2^{C120Y/-}$; and *fl/fl*, $Ovol2^{fl/fl}$.

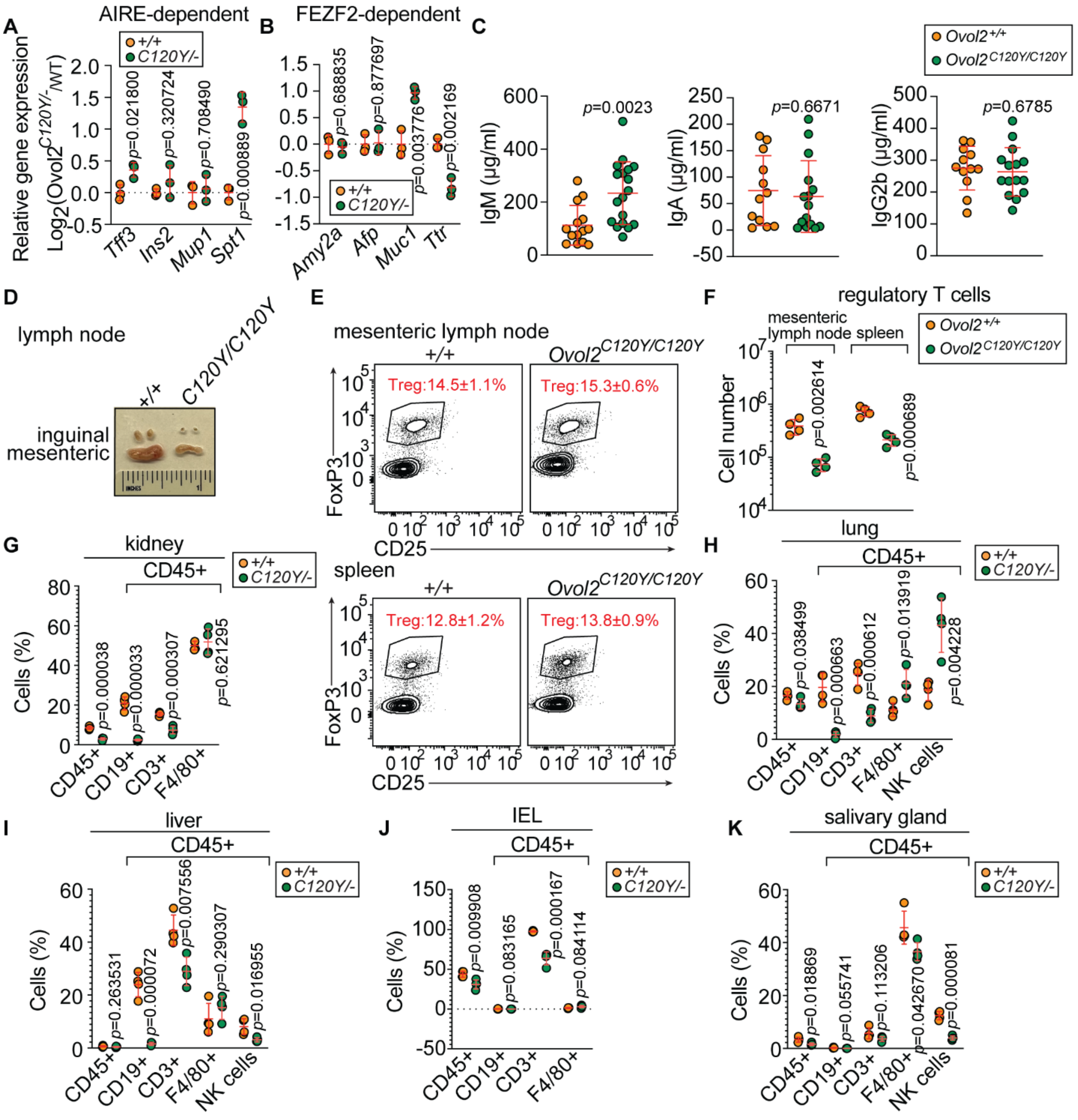

Supplementary Figure 11. *Ovol2*$^{C120Y/-}$ mice showed normal TRA expression and did not display signs of autoimmunity.

(**A-B**) Real time-PCR analysis of the indicated AIRE (A) or FEZF2 (B) dependent tissue-restricted self-antigens (TRAs) in FACS sorted MHC-II^{high} mTECs from 12-wk-old *Ovol2*$^{C120Y/-}$ thymi and WT littermates (*n* =3 per group). (**C**) Serum IgM, IgA, and IgG2b detected in *Ovol2*$^{C120Y/C120Y}$ and *Ovol2*$^{+/+}$ littermates by ELISA (*n* =18 *C120Y/C120Y*, 14 WT littermates for IgM, 15 *C120Y/C120Y*, 12 WT littermates for IgA and IgG2b). Data presented as absorbance at 450 nm. (**D**) Representative photographs of inguinal or mesenteric lymph nodes from 20-wk-old *Ovol2*$^{C120Y/C120Y}$ and WT littermates. (**E** and **F**) Representative flow cytometry plots (E) and quantitative analysis (F) of FoxP3+ regulatory T cells from mesenteric lymph node or spleen in

20-wk-old *Ovol2*$^{C120Y/C120Y}$ mice and WT littermates. Numbers adjacent to outlined regions indicate percent cells in each (mean ± SD) (*n* =4 mice/genotype). (**G**-**K**) The frequency of lymphocytic infiltration and macrophages (F4/80+) in kidney (G), lung (H), liver (I), intestine (J), and salivary gland (K) of *Ovol2*$^{C120Y/-}$ and WT littermates at 12 wk of age (*n* =4 mice/genotype). Data are representative of one experiment (C) or two independent experiments (A-B and E-K). Data points represent individual mice (C, F-K) or individual sorting pools from 10 mice per pool (A-B) and means ± SD are indicated. *P* values were determined by two-tailed Student's *t* test. IEL: intestinal intraepithelial lymphocytes. +/+, WT; *C120Y/-*, *Ovol2*$^{C120Y/-}$; *C120Y/C102Y*, *Ovol2*$^{C102Y/C120Y}$.

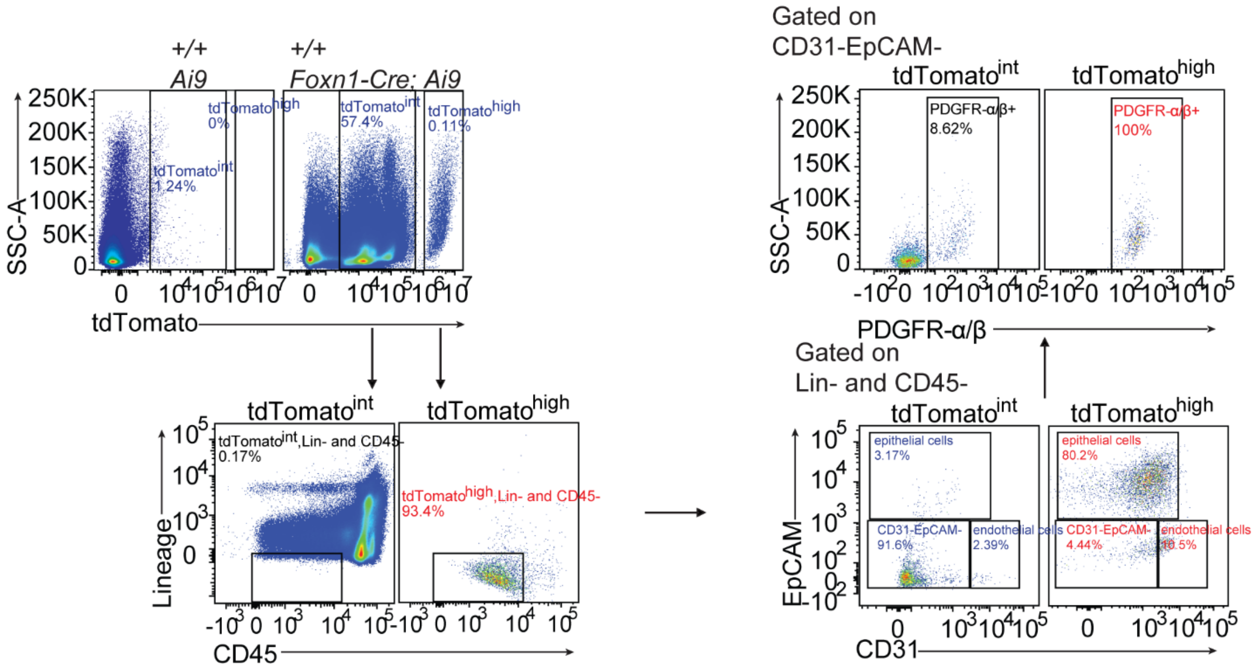

Supplementary Figure 12. Flow cytometry gating strategy for lineage tracing of tdTomato^{high} cells in the thymus of *Foxn1-Cre*;*Ai9* mice.

Foxn1-Cre;*Ai9* mice express Ai9, a Cre reporter knocked into the *Rosa26* locus, which contains the *tdTomato* gene separated from a CAG promoter by a loxP-flanked STOP cassette. CAG promoter-driven expression of tdTomato is activated by Cre-mediated deletion of the STOP cassette. Driven by the *Foxn1* promoter, Cre expression activates tdTomato fluorescence in TEC progenitors. The population of tdTomato^{int} cells in *Foxn1-Cre*;*Ai9* thymi was found to be Lin+CD45+ (marking cells of hematopoietic origin) and were thus excluded from analysis. tdTomato^{high} cells were Lin-CD45-, the majority of which were epithelial cells as expected; this population was analyzed in Fig. 5C-G. Numbers adjacent or inside outlined regions indicate representative percentage in each population. +/+, WT.

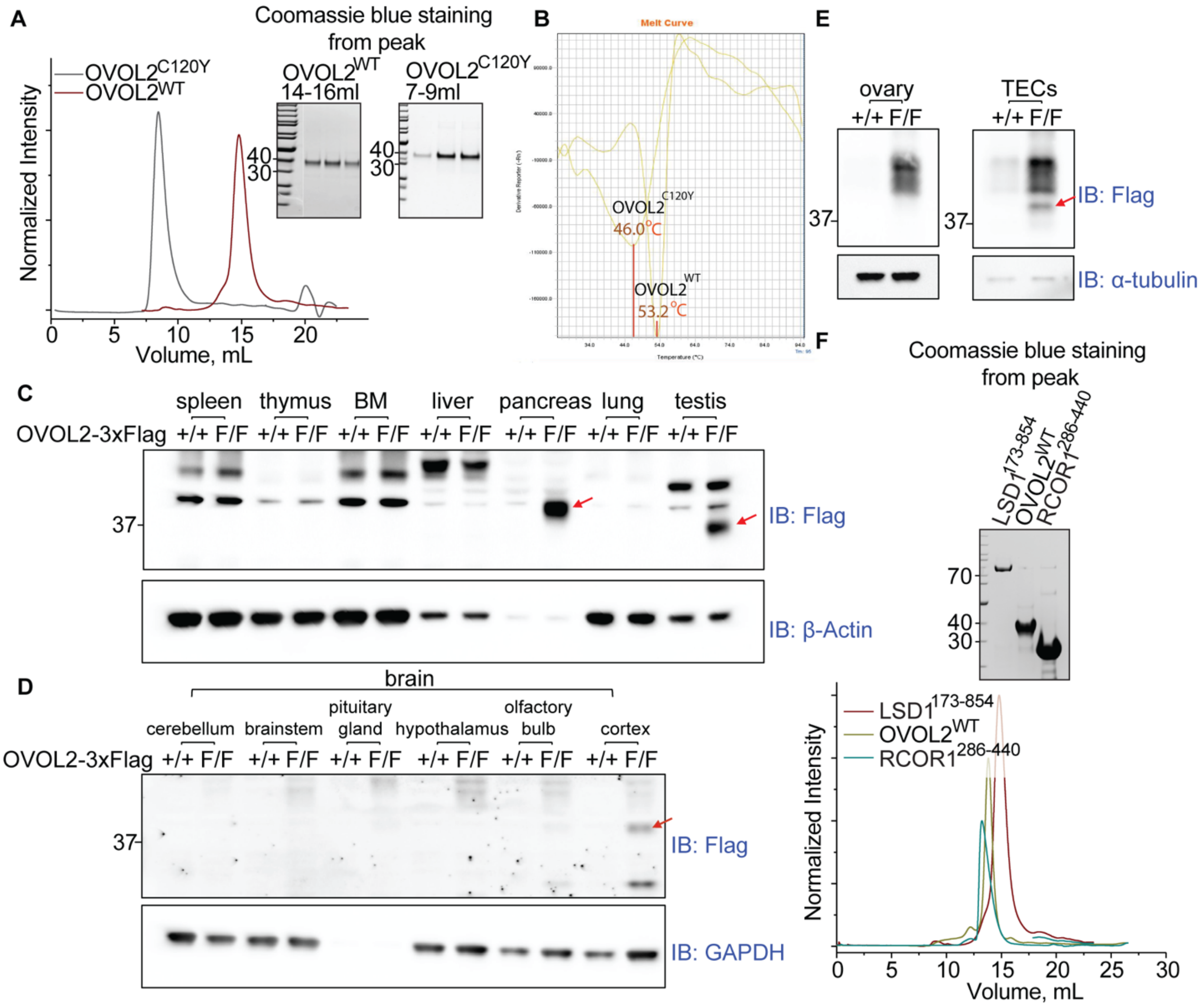

Supplementary Figure 13. Biochemical properties of OVOL2^{C120Y} and tissue expression of OVOL2-3xFlag.

(**A**) Sepharose gel filtration chromatography of pure OVOL2^{WT} or OVOL2^{C120Y} protein. (**B**) Reduced thermal stability of OVOL2^{C120Y}. Thermal stability of OVOL2^{WT} and OVOL2^{C120Y}. The temperature at the lowest point of each curve is the Tm. OVOL2^{WT} and OVOL2^{C120Y} have a Tm of 53.2°C and 46°C, respectively. (**C-E**) OVOL2 is expressed in pancreas, testis, cortex of brain, and TECs in thymus. Immunoblot analysis of OVOL2-3xFlag in various tissues from WT or *Ovol2*$^{F/F}$ mice, including spleen, thymus, bone marrow (BM), liver, pancreas, lung, testis, brain (cerebellum, brainstem, pituitary gland, hypothalamus, olfactory bulb, and cortex), ovary, and FACS sorted TECs. (**F**) Sepharose gel filtration chromatography of purified OVOL2^{WT}, LSD1$^{173-854}$, or RCOR1$^{286-440}$ protein. Data are representative of three independent experiments. +/+ indicates WT and *F/F* indicates the *Ovol2*$^{3xFlag/3xFlag}$ genotype.

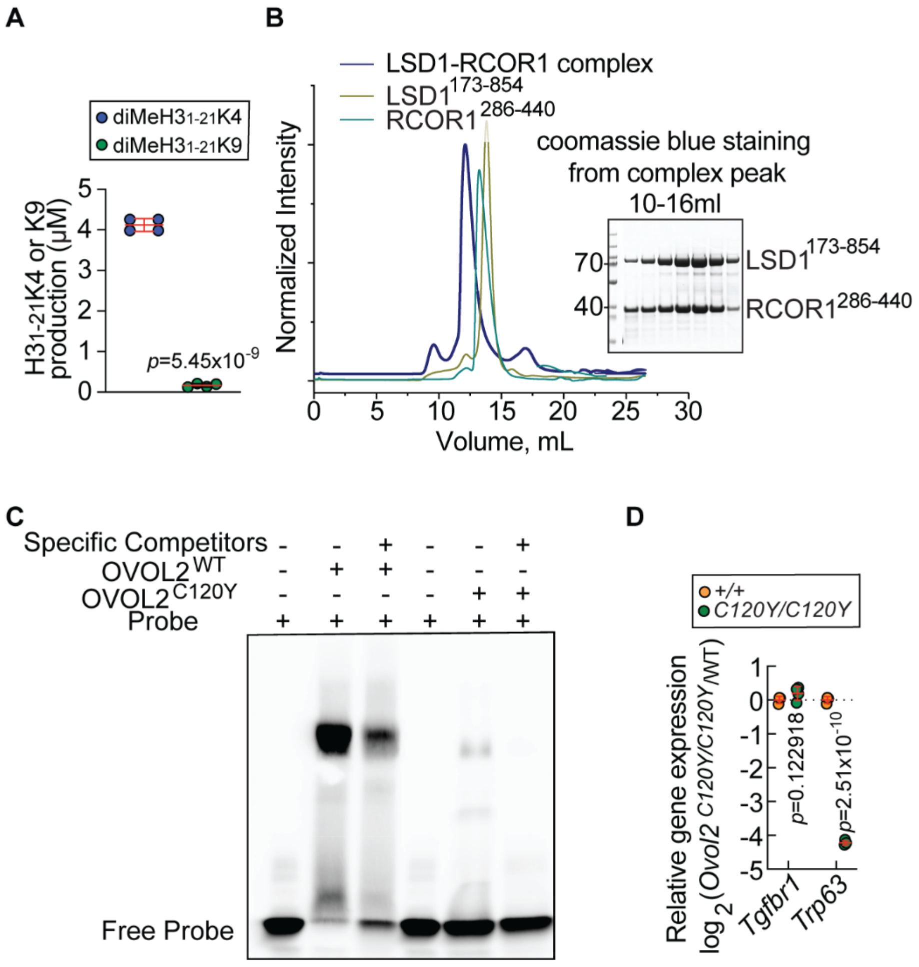

Supplementary Figure 14. (**A**) ***In vitro*, LSD1 does not demethylate diMeH3$_{1\text{-}21}$K9.** LSD1 demethylation activity assay. Demethylation activity of purified LSD1 (300nM) towards 60μM diMeH3$_{1\text{-}21}$K4 or diMeH3$_{1\text{-}21}$K9 substrate (*n* =4 wells per condition). (**B**) **LSD1-RCOR1 form a complex.** Sepharose gel filtration chromatography of LSD1-RCOR1 complex or LSD1$^{173\text{-}854}$ or RCOR1$^{286\text{-}440}$. (**C**) ***C120Y* mutation impaired OVOL2 DNA binding.** EMSA analysis of the effect of the *C120Y* mutation on OVOL2-DNA binding. 1.7 μg of purified OVOL2^{WT} or OVOL2^{C120Y} was incubated with OVOL2 probe (AACCGTTACC) (1nM) with or without specific competitor (4.5μM) for 30min before being harvested for EMSA analysis. (**D**) Real time-PCR analysis of *Tgfbr1* and *Trp63*, which were accessible in both *Ovol2*$^{+/+}$ and *Ovol2*$^{C120Y/C120Y}$ TEC chromatin, but in different regions (*n* =4 *C120Y/C120Y* wells, 3 WT wells). Data are representative of two independent experiments. Data points represent individual sorting pools from 5 mice per pool and means ± SD are indicated (D). *P* values were determined by two-tailed Student's *t* test (D). +/+ indicates WT and *C120Y/C120Y* indicates the *Ovol2*$^{C120Y/C120Y}$ genotype.

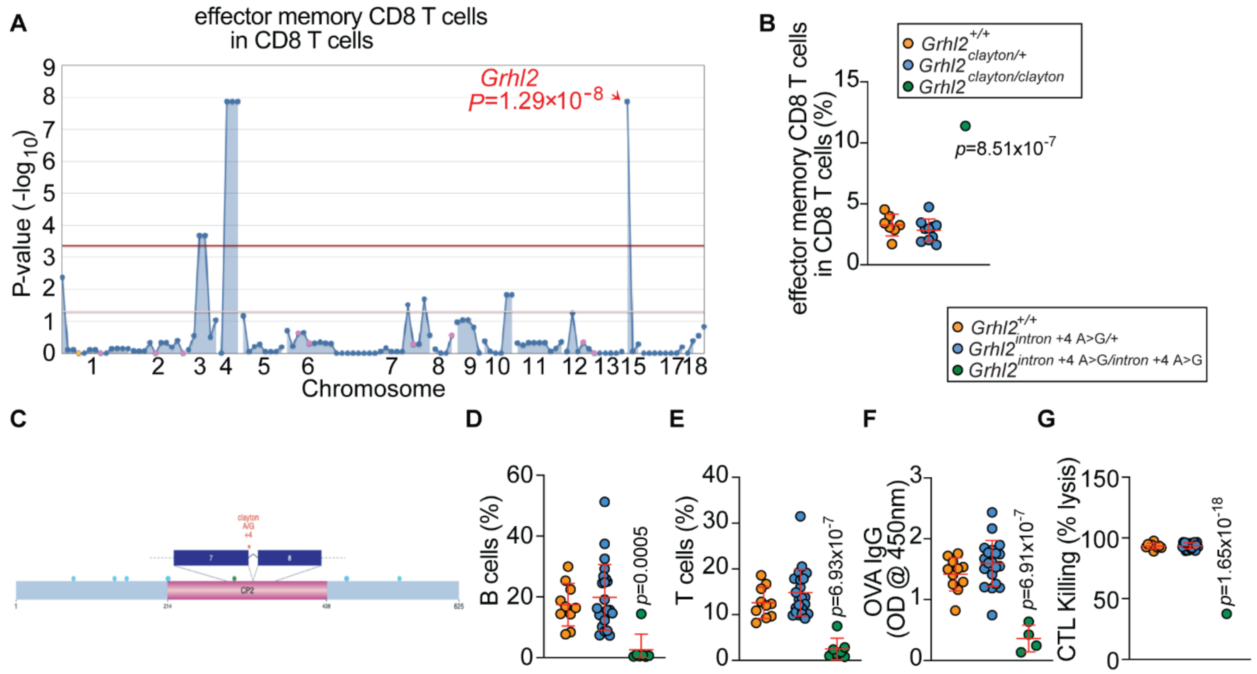

Supplementary Figure 15. Reduced frequencies of peripheral B cells and T cells, impaired T-dependent antibody responses to immunization, and defective CD8 T cytolytic function caused by a *Grhl2* mutation.

(**A**) Manhattan plot. -Log_{10} *P* values plotted *vs.* the chromosomal positions of mutations identified in the G1 founder of the affected pedigree. (**B**) The frequency of effector memory CD8 T cells in peripheral blood CD8 T cells from third generation (G3) descendants of a single ENU-mutagenized male mouse, with REF (+/+), HET (+/*clayton*), or VAR (*clayton/clayton*) genotypes for *Grhl2* (*n* =1 $Grhl2^{clayton/clayton}$, 10 $Grhl2^{clayton/+}$, 7 $Grhl2^{+/+}$ littermates). (**C**) GRHL2 topology. Numbers are amino acid positions. A single nucleotide change (A to G) at the fourth nucleotide of *Grhl2* intron 7 is predicted to result in skipping of exon7 during splicing. The resulting *Grhl2* transcript would have a 112-nt deletion of exon7, and encode a frame shifted protein product beginning after amino acid 297 of the protein, which is normally 625 amino acids in length, and terminating after the inclusion of 26 aberrant amino acids. (**D** and **E**) The frequency of peripheral blood B cells (D) and T cells (E) from 10-week-old *intron* +4 A>G/ *intron* +4 A>G mice generated by the CRISPR/Cas9 system (*n* =7 $Grhl2^{intron+4A>G/\ intron+4A>G}$, 23 $Grhl2^{\ intron+4A>G/+}$, 10 $Grhl2^{+/+}$ littermates). (**F**) Serum ova-specific IgG level in mice immunized with T cell-dependent antigen alum-ova (*n* =4 $Grhl2^{intron+4A>G/\ intron+4A>G}$, 20 $Grhl2^{\ intron+4A>G/+}$, 11 $Grhl2^{+/+}$ littermates). Data presented as absorbance at 450 nm. (**G**) Quantitative analysis of the Ova-specific cytotoxic T cell killing response in mice immunized with alum-ova (*n* =1 $Grhl2^{intron+4A>G/\ intron+4A>G}$, 20 *Grhl2* $^{intron+4A>G/+}$, 10 $Grhl2^{+/+}$ littermates). Data are representative of one experiment (B and D-G). Data points represent individual mice and means ± SD are indicated. *P* values were determined by one-way analysis of variance (ANOVA) with Dunnett's multiple comparisons (B and D-G). CTL, cytotoxic T lymphocyte.

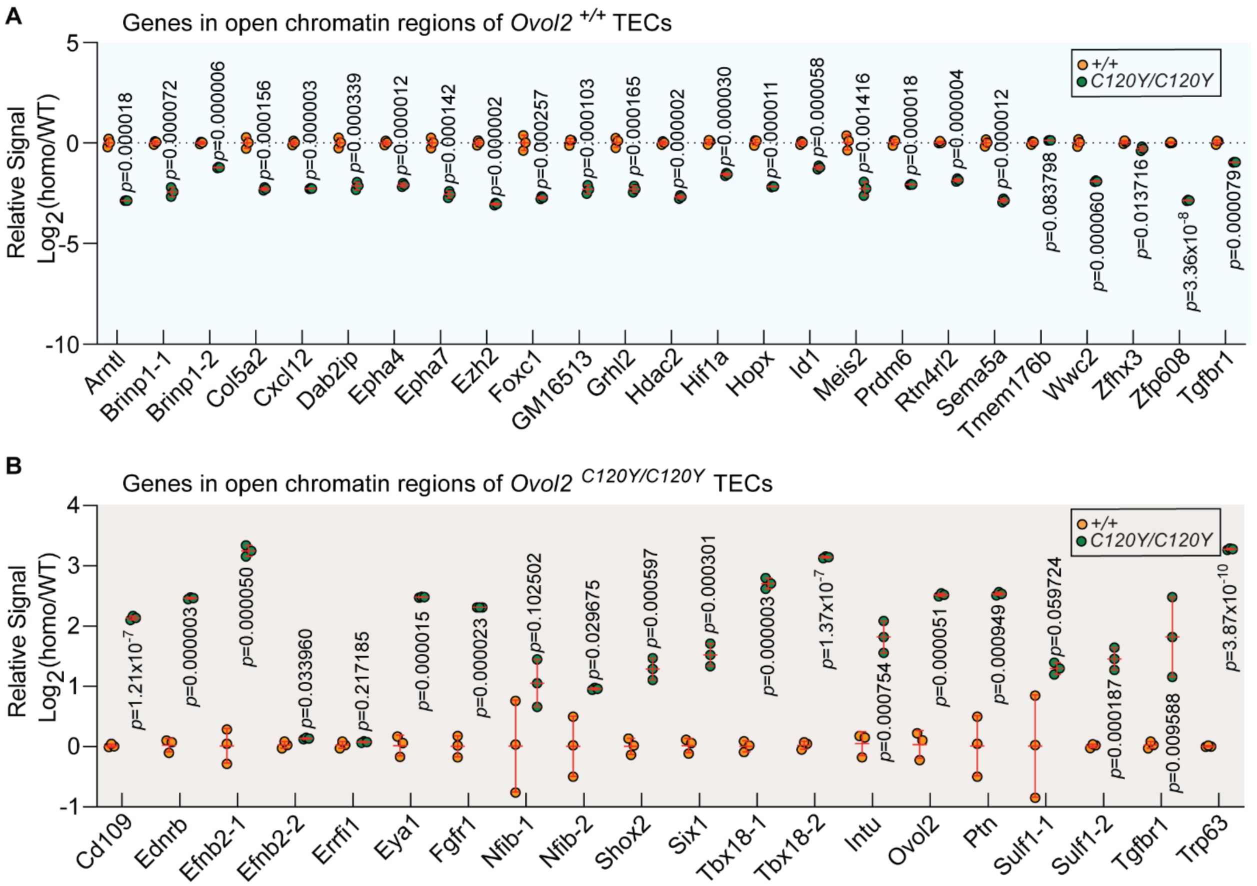

Supplementary Figure 16. Chromatin immunoprecipitation (ChIP) analysis of selected genes associated with H3K4me2 in WT and *Ovol2*$^{C120Y/C120Y}$ TECs by CUT&RUN assay.

CUT&RUN assay was performed using H3K4me2 antibody and TECs isolated from 4.5-wk-old WT or *Ovol2*$^{C120Y/C120Y}$ mice. Precipitated DNA representing 45 loci with altered chromatin configuration and gene expression in *Ovol2*$^{C120Y/C120Y}$ TECs was measured by real time-PCR. Data were normalized to *Rpl30*. (**A**) 25 loci accessible in *Ovol2*$^{+/+}$ TECs but not in *Ovol2*$^{C120Y/C120Y}$ TECs (*n* =3 per group). (**B**) 20 loci accessible in *Ovol2*$^{C120Y/C120Y}$ TECs but not in *Ovol2*$^{+/+}$ TECs (*n* =3 per group). Data are representative of two independent experiments. Data points represent individual sorting pools from 5 mice per pool and means ± SD are indicated. *P* values were determined by two-tailed Student's *t* test. +/+ indicates WT and *C120Y/C120Y* indicates the *Ovol2*$^{C120Y/C120Y}$ genotype.

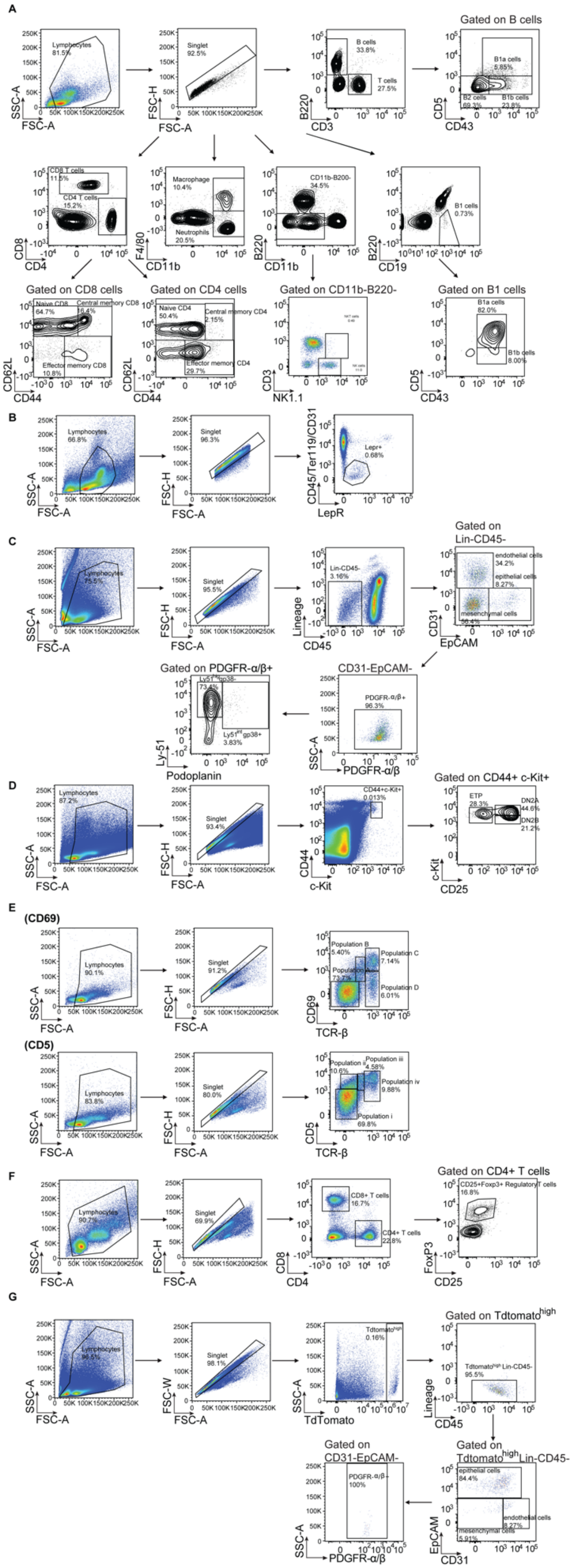
A
Gated on B cells
Lymphocytes 81.5%
Singlet 92.5%
B cells 33.8%
T cells 27.5%
B1a cells 5.85%
B2 cells 69.3%
B1b cells 23.8%
SSC-A
FSC-A
FSC-H
B220
CD3
CD5
CD43
CD8 T cells 11.5%
CD4 T cells 15.2%
Macrophage 10.4%
Neutrophils 20.5%
CD11b-B220- 34.5%
B1 cells 0.73%
CD8
CD4
F4/80
CD11b
CD19
Gated on CD8 cells
Gated on CD4 cells
Gated on CD11b-B220-
Gated on B1 cells
Naive CD8 64.7%
Central memory CD8
Effector memory CD8 10.8%
Naive CD4 50.4%
Central memory CD4 2.15%
Effector memory CD4 29.7%
B1a cells 82.0%
B1b cells 8.00%
CD62L
CD44
NK1.1
B
Lymphocytes 66.8%
Singlet 96.3%
Lepr+ 0.68%
CD45/Ter119/CD31
LepR
C
Lymphocytes 75.5%
Singlet 95.5%
Lin-CD45- 3.16%
Gated on Lin-CD45-
endothelial cells 34.2%
epithelial cells 8.27%
mesenchymal cells
Lineage
CD45
CD31
EpCAM
Gated on PDGFR-α/β+
CD31-EpCAM-
PDGFR-α/β+ 96.3%
Ly51
Podoplanin
PDGFR-α/β
D
Gated on CD44+ c-Kit+
Lymphocytes 87.2%
Singlet 93.4%
CD44+c-Kit+ 0.013%
ETP 28.3%
DN2A 44.6%
DN2B 21.2%
CD44
c-Kit
CD25
E
(CD69)
Lymphocytes 90.1%
Singlet 91.2%
Population B 5.40%
Population C 7.14%
Population D 6.01%
CD69
TCR-β
(CD5)
Lymphocytes 83.8%
Singlet 80.0%
Population iii 4.58%
Population iv 9.88%
Population i 69.8%
CD5
F
Gated on CD4+ T cells
Lymphocytes 90.7%
Singlet 69.9%
CD8+ T cells 16.7%
CD4+ T cells 22.8%
CD25+Foxp3+ Regulatory T cells 16.8%
FoxP3
G
Gated on Tdtomato^high
Lymphocytes 96.5%
Singlet 98.1%
Tdtomato^high 0.16%
FSC-W
TdTomato
Gated on CD31-EpCAM-
PDGFR-α/β+ 100%
Gated on Tdtomato^high Lin-CD45-
epithelial cells 84.4%
endothelial cells 8.27%
mesenchymal cells 5.91%

Supplementary Figure 17. Flow cytometry gating strategies.

(**A**) Lymphocytes and myeloid cells in secondary lymphoid tissues and other tissues. Corresponds to Figs. 1A, 1B, 1D, 1E, 1H (CD4+ and CD8+), 1I-K, 2A, 2B, 2E, 2F, 2I-M, and Supplementary Figs. 2H, 11G-K, 15A-B, 15D-E. (**B**) LepR+ cells in bone marrow. Corresponds to Supplementary Fig. 3. (**C**) Endothelial and mesenchymal cells in thymi. Corresponds to Figs. 4C, 4F, 4H, 4K, 4M, 4P, and Supplementary Fig. 7. (**D**) T-cell progenitors (ETP) in thymi. Corresponds to Figs. 4D, 4I, 4N, and Supplementary Fig. 8. (**E**) Thymocytes undergoing positive selection. Corresponds to Supplementary Fig. 9. (**F**) Regulatory T cells (Treg cells). Corresponds to Supplementary Fig. 11E-F. (**G**) Epithelial, endothelial, and mesenchymal cells in the thymus of *Foxn1-Cre*;*Ai9* mice. Corresponds to Fig. 5C-F and Supplementary Fig. 12.

Table S1. Primer sequences

Gene	Forward primer	Reverse Primer
boh_pcr	5'-TGCTCCACACTGCTAACTTG-3'	5'-AGCCTACGTGCACCTTAGAG-3'
boh_seq	5'-CACACTGCTAACTTGCTTTGGGG-3'	5'-TACGTGCACCTTAGAGGGAGC-3'
OVOL2-3xFlag-TG	5'-TGAGGTGTGTAACAAGGCCTT-3'	5'-CTTGTCATCGTCATCCTTGTAATCGATATCATGATC-3'
OVOL2-3xFlag-CS	5'-TGAGGTGTGTAACAAGGCCTT-3'	5'-CCAGGGGAGCAGAGGGGTACG-3'
Bmp4	5'-TATCAGGAGATGGTGGTAGAGG-3'	5'-AGGTCAAGGTGAGTTGTTTAGG-3'
Cav2	5'-GGGCAACTTCCTCAGACATTAG-3'	5'-CCAGATGAAGCCCAGTCATAAA-3'
Cd109	5'-CTGAGTGAGACCCTGAAGAAAG-3'	5'-CAGATGCTGAAGGACTGGATAC-3'
Cnmd	5'-CTCTGAACTGGAAGGCAAGAT-3'	5'-CTGCTGGTGGTAAGGATTGT-3'
Ednrb	5'-GATGAGCGGTGTGTGAAAGA-3'	5'-CCCTTGGTCTGTGTGCTAAA-3'
Efnb2	5'-CGGGTGTTACAGTAGCCTTATT-3'	5'-GCTGCTCGGATCTCATTTCT-3'
Errfi1	5'-GCGGTGTAACCATAGACCATAG-3'	5'-CCAGCTCGTGTTGTCCTTTA-3'
Eya1	5'-GTGTGTGTGTGTGTGTGTATTG-3'	5'-GGTGGGAAGGAAAGAGTAAGAG-3'
Fgfr1	5'-GAGGGTAGAACTGGACAGAAAC-3'	5'-GACCAACCAACCAACCAAAC-3'
Fgfr2	5'-CCAGCACTGGAGCCTTATTAT-3'	5'-GATCCTCTGGCAACTCATACTC-3'
Gas1	5'-AGAAGGGATGTTGAGGGAATTAG-3'	5'-AGCTCCACACTGAGCATTAC-3'
Gata3	5'-CAGTCCTCATCTCTTCACCTTC-3'	5'-GGTCTGGATGCCTTCTTTCT-3'
Gja1	5'-TGTCCCACCTTTGTGTCTTC-3'	5'-CTGCCTCTGCTTGTACCAATA-3'
Ift57	5'-GCCTGGTGGCTCATTAGTATTT-3'	5'-GGGTATCTGTCTGTCTCTCTGT-3'
Intu	5'-GCTGACCATGCCCGTAAATA-3'	5'-CCACCAAGCCAGACATAACA-3'
Irs2	5'-CCAGAACGGCCTCAACTATATC-3'	5'-CCTGCCTCTTGGTTCCTTATC-3'
Jun	5'-CCAACCTCTTTGCTGCATTATC-3'	5'-CAACCAAAGTGTCTGCTTTCC-3'
Kdr	5'-TCTGTCAAGTGGCGGTAAAG-3'	5'-GATGAGAGAGACGGTAGGGTAT-3'
Lep	5'-TGAGAGGACACTTGATGGAAAG-3'	5'-TTGAGTGCTGGCATGAGTAG-3'
Lrp6	5'-GAAGATGGGAGGGTAGCAATAC-3'	5'-CTAAGACGTACCCTGAGCAATC-3'
Mcc	5'-CCTTCCTTCCTTCCTTCCTTTC-3'	5'-GGACTCTACAACCAACCTCTTC-3'
Mef2c	5'-TTGTGACCAGTCACGTTCTATC-3'	5'-GACACCAGCATTCAAGCAATC-3'
Muc16	5'-CCATCACCAACCTACCCTATTC-3'	5'-CAGGTCCAGGTGAGACAAATAA-3'
Nfib	5'-GAAAGTGGAGCAGAGGGAAATA-3'	5'-TTGGCTGAGAGGATGTGTTATC-3'
Nr4a3	5'-TGGAGGCTGAGATACAGAGTAA-3'	5'-AAGGCTGTCTGCTGGTAAAG-3'
Ovol2	5'-TCAGTCCACCTCTCTCTTCTT-3'	5'-CCTTGCATGGGAAGCATTTC-3'
Pex2	5'-GTACAGCCACTCTCTTCCTTTAC-3'	5'-CTTAGTTACCAGCGAGACGTTAG-3'
Phf14	5'-CAGGCATGTGTAGAGCCTATTT-3'	5'-CTCTGCCTTGGCACGATATT-3'
Ptn	5'-GGCCGGGAAGAAAGAGAAA-3'	5'-CAAGCCTGGAACTGGTACTT-3'
Ptprk	5'-GAGTACCTGGAGTCCTCATAGT-3'	5'-CAGACACAGACCACTTCTCATC-3'
Shox2	5'-CGGATCTCAGACTGAAAGCTAAA-3'	5'-GATCCAGGGTGCAGAAAGAA-3'
Six1	5'-TCCTCCTCCTCGTCTTCTTT-3'	5'-GCCTCCCATAGAGAGCATTAAG-3'
Smo	5'-CTGCCTAGAAGAGCTGTGTATG-3'	5'-CTGTGTGTGTGAGAGTGTATGT-3'
Sulf1	5'-TGTCTGCTTGGTTGGTAGTG-3'	5'-GCCGGTCATTCTTGCTTTATTT-3
Tbx18	5'-CAGAGGGTTATCTCCAGCAATC-3'	5'-CTTACAGAATCCGCAGGGAAA-3'
Tgfbr1	5'-CATGGGAGTGTTCTGGTTCTAC-3'	5'-CCTGGTGGATGCTGACAAATA-3'
Thbs1	5'-GAGGAGCTGTTCGGTACTAAAT-3'	5'-GAGTACTCCTGGCTTCCATTAC-3'
Tnfaip3	5'-GGAGCCCTCATTCTGGTTTATT-3'	5'-CAGCCGATTCTACAGGCTTATC-3'
Trp63	5'-GGTGGTGAAGCAGATAGGATAG-3'	5'-GCTCACCATAGCCCATAACA-3'
Zfp36l1	5'-CACAGGTAGGACTGAGAATGTAT-3'	5'-GTGTGCGCCAGTTGTTTATG-3'
Adgrv1	5'-GTTTCCTTCTCTGTGGGTTCTC-3'	5'-CTACTCGCAGGTTGAGGTTATG-3'
Arntl	5'-TGCCACTGACTACCAAGAAAG-3'	5'-CAAACAAGCTCTGGCCAATAAG-3'
Brinp1	5'-CACAGGGTGGTCAGTTCTATTC-3'	5'-GTGTCTGTCTGTCTGTCTGTATG-3'
Cib1	5'-TGCATGGTCTTCTCCACATC-3'	5'-CCCTGTCAATGTCTGACTCTTC-3'
Clec2d	5'-TGGAAGTGGACAGACAACAC-3'	5'-GAAAGAGTAGCACCCACAGATAA-3'
Col3a1	5'-GCCTCCCAGAACATTACATACC-3'	5'-CAGCAGAAGAGAAGCACCTTTA-3'
Col5a1	5'-CGTGCCTTTCCGATGGATTA-3'	5'-TGGCACACACACAGAGATTAG-3'

Col5a2	5'-AAGTGCCTGACCACCTTATC-3'	5'-GTGACTGTGGGAGAGTCTTAAC-3'
Dab2ip	5'-GATGGACAGGCAAGGGTAAA-3'	5'-GCACCAGGACTAAAGGACTATG-3'
Dlx1	5'-GTGTCTGCTGTCCTCATTCTAC-3'	5'-GTTCCCTCTTCTGGTGTGTTTA-3'
Enpp1	5'-CCCTGTCATTCTGGCATCTT-3'	5'-GCCTGTTTCCTCGGGATTATAG-3'
Epha4	5'-CCAGTGGCTCTTGAGGTTAAT-3'	5'-GGTGAAACGGCTTGATTTGG-3'
Epha7	5'-TAGCAAGGCTGACCAAGAAG-3'	5'-CCCTGTGAACGTATCCCATATC-3'
Erbb4	5'-GGGATCACCTGGTCACAAATAG-3'	5'-AGAGAAGCCCACAGTGATAGA-3'
Ezh2	5'-CAGGATGAAGCAGACAGAAGAG-3'	5'-AGCTGGTGAGAAGGCAATAAA-3'
Foxc1	5'-GAAGCAAATGTACCCGGAGATA-3'	5'-TCTAGATAGGAGCGGCAGATAG-3'
Gm10424	5'-GCACTCCTACCTTCCTTCATAC-3'	5'-CCTGACAAAGTTGGGCAAATC-3'
Gm16513	5'-GGGCATGATGGAGTCAGAATAA-3'	5'-GGCAAGAGACTAGGGAAAGATG-3'
Gm3183	5'-GGCACCACTCCACAAGAATA-3'	5'-GGCAGGTAAGAGGACAATGAG-3'
Gm6509	5'-CCTTGAAGACCCTCTCCATTAC-3'	5'-CTGGGAGTCCTTCATTCTACAC-3'
Grem1	5'-GTAACTCTGAGGGCTGCATTAG-3'	5'-CTCTGTGTGTCTGGGTTCTTT-3'
Grhl2	5'-GGAGATAGCAAGTCCACTCATC-3'	5'-GGCATACTCGGGTTCCTTATT-3'
Hdac2	5'-TGGAGATGAGGATGGAGAAGA-3'	5'-CGAGGTTCCTAAAGTTGGAGAG-3'
Hif1a	5'-GCCCACCCTGTTGGTATAAA-3'	5'-TAGCTTCCTTCACCTGCTAATG-3'
Hopx	5'-GAACTCCATCTCTTCTTCCTTCC-3'	5'-GACGGTTCCCACATCATCTT-3'
Id1	5'-GGAGATCCTGCAGCATGTAAT-3'	5'-CAAAGTCTCTGGAGGCTGAAA-3'
Meis2	5'-CCCTTCCCTGTTTCCTTGTTAG-3'	5'-GGGAGGGAGGCTCTATGTATTT-3'
Mapk11	5'-GAGAGGTGCCAAGATCCATTAG-3'	5'-AGGGTTGGTGGAGGTAGAA-3'
Pik3r1	5'-AACAGCAACTCCAGACCTAATG-3'	5'-AGGGACTACCATCAACCTCTAC-3'
Plg	5'-CAGCCACCATCACGGATAAA-3'	5'-GAACGTAGACACCAGGCTTATT-3'
Prdm6	5'-GTCAGACCAGGCAAGAGATTAT-3'	5'-ACAGGAGGAAGGCCATAGA-3'
Rtn4rl2	5'-GCTCCATCTACAGGATGACTTG-3'	5'-GTTGAACAGGTAGAGGATGGTG-3'
Ryk	5'-CCACAGAAACCTCCTTCCTATT-3'	5'-AGTTGATTGGCTGGGCTATT-3'
Sema5a	5'-GACCCTCCTCTCTCATCCTAAT-3'	5'-TCACTCCCTGTGCTCTATATCT-3'
Sox17	5'-CTACACACAACCTCCAGCTTTA-3'	5'-ACCTCGCCTTTCACCTTTAC-3'
Tgfbr1	5'-CATGGGAGTGTTCTGGTTCTAC-3'	5'-CCTGGTGGATGCTGACAAATA-3'
Tmem176b	5'-GCTGTGCTCTTGGAGTATGT-3'	5'-GGAGAGAAAGAGGGCTGAATAG-3'
Trp63	5'-GGTGGTGAAGCAGATAGGATAG-3'	5'-GCTCACCATAGCCCATAACA-3'
Wwc1	5'-TGAAGGCTGACTGTGACTAATG-3'	5'-GCAGGGAAACCTCTGAAAGT-3'
Wwc2	5'-CTCACAAGGTGTAACTGGATAGG-3'	5'-CTACAGAAAGCGGCCTGTATAA-3'
Zfhx3	5'-CCTTGACCCACGAGTGTATTT-3'	5'-ACCACTGACTGCTTCACTATTT-3'
Zfp36l2	5'-CTGCCACCTCCCTAAACTATAAG-3'	5'-CTTCCAGAGAGCAGAGGAAATG-3'
Zfp608	5'-CATCAAAGAGGAGCCCAAAGA-3'	5'-CCAGGGTAACTGTGCATTAAGA-3'
Zfp706	5'-ACAGTCAGTGTGAGTGCTTTAT-3'	5'-GAGACAGAGCAAGGTCCTTATG-3'
DLL4	5'-GCCTTCCTTCTGCATTGTTTAC-3'	5'-CCTCCTCTCTGCTTTCTCATTT-3'
IL7	5'-GCAGGAGACTAGGACCCTATAA-3'	5'-GAGCAGGCATCGGTCAAATA-3'
SCF	5'-TGGGAGAAACCAAGCCTATAAC-3'	5'-CCAACTACACCTAACTGCCTAC-3'
CXCL12	5'-GCTAAGGTTTGCCAGCATAAAG-3'	5'-CCTGGCACTGAACTGGATAAA-3'
PSMB11	5'-GTTGTTCTGTGGAAGAGAGAGG-3'	5'-ATGGAAGGAAGAGAGAGGAGAG-3'
CATHEPSINL	5'-TAGCTCAGTGTGTGTGTATGTG-3'	5'-CCCAGCCTGACAACCTTATT-3'
PRSS16	5'-TAGTGGACTTCTCCGCCTATAA-3'	5'-ACCCTCACAGGTGACATAGA-3'
Foxn1	5'-CTATGCCACTCAGCCAACTTAT-3'	5'-CATGATGAGCAGGTGTGTAGAG-3'
CD83	5'-CCAAACACAGGAGGCTACAA-3'	5'-AAAGTCATGGCTGAGAGGTAAG-3'
CCL19	5'-CGCATCATCCGAAGACTGAA-3'	5'-CTCTCTTGTCCACACTCACATC-3'
CCL21	5'-GTCCAACTCACAGGCAAAGA-3'	5'-TCATAGGTGCAAGAACAAGGG-3'
P-selectin	5'-GGCATCCATACTCACAGGTAAA-3'	5'-GGGTGGCTCAAGTCTGTAATG-3'
CCL25	5'-GGAAGGAACTACTGCGCTAAA-3'	5'-GCACTCTCACACACTCACTATC-3'
Ovol2	5'-TCAGTCCACCTCTCTCTTCTT-3'	5'-CCTTGCATGGGAAGCATTTC-3'
Snai1	5'-CTTCAGGCCACCTTCTTTGA-3'	5'-ATAGTTCTGGGAGACACATTGG-3'
Cxadr	5'-AGCTAAACACTGCCCTTCTAC-3'	5'-CTACACACACTCCCTCACAATC-3'
Cldn12	5'-GTCTCCTCCCATAGCCGTAATA-3'	5'-GACCAACACTCAGCTCCATTTA-3'

Krt18	5'-CCAGACAAGATGAGCTTCACA-3'	5'-CCAGTCTCCTGTTCTCAGTTTC-3'
Cdh4	5'-CAAGTGGCCGGTGAGATTAT-3'	5'-TGTCTGAGGAGTGAGAGTAGAC-3'
GAPDH	5'-ACTCCACTCACGGCAAATTC-3'	5'-TCTCCATGGTGGTGAAGACA-3'
bAct	5'-CATTGCTGACAGGATGCAGAAGG-3'	5'-TGCTGGAAGGTGGACAGTGAGG-3'
Arntl	5'-GGTATTGCTGCTCTCCAGTT-3'	5'-TGGGCTTTATTTGGTTTGGTTT-3'
Brinp1-1	5'-CTGGGATCGGAAAGGTGATT-3'	5'-AGATGAATAGAAAGAGAGGGCTAA-3'
Brinp1-2	5'-CCATAAGTGCCTCTCAGCTTTA-3'	5'-CTCCACCACATCTTCATCTCTG-3'
Col5a2	5'-GTAACCACTCACATGGGTACAT-3'	5'-TCAAATTGCTTCACACTCAAAGG-3'
Cxcl12	5'-CCAGTTCAGACAGAAGGAGAATAA-3'	5'-ATAGAAGCCCACAGTGCATC-3'
Dab2ip	5'-GCAACCTCAATAGGTGGTATCT-3'	5'-CTTACAGCTAACTCCCGTGAC-3'
Epha4	5'-GGGAAGGAACCCAAGATGAAA-3'	5'-TGGATGTAGGTGGAGTCAGTAA-3'
Epha7	5'-CCGGCACACTGTAGAACATAG-3'	5'-AGTTCACTCATGCAGTATCTTGA-3'
Ezh2	5'-GGGTGAGTGTGTAAGAAGTGTG-3'	5'-GTTGTGTCGTCATCGTTGGTA-3'
Foxc1	5'-GTAGAGTGCTCATGGCGTTAT-3'	5'-TTGTGTCTACCAGAGTCCAAAG-3'
GM16513	5'-GTGCATGGGACAAGGAGTATAA-3'	5'-GCCAGGACTACACAGAGAAAC-3'
Grhl2	5'-CTACAACACTCAGGAGCAAGTC-3'	5'-CCTTTAACACCAGGACCAACA-3'
Hdac2	5'-ACCTAGCCCAAGCTGATTTC-3'	5'-CATGTGTTTGGCTACGTTCATC-3'
Hif1a	5'-CAACTCACCAAGAACCTCTCAA-3'	5'-TGAGCTATATCCCAGCCTCTAC-3'
Hopx	5'-TGCCCATCTAGCTTGAACAG-3'	5'-CTGTCAGATGGGTTTCCTAGTG-3'
Id1	5'-CGAGGAATGCGGAGCTATTTA-3'	5'-CAACACATACACACACACACAC-3'
Meis2	5'-GGGACTTTGTTCTTCCGTTCT-3'	5'-GGCCACAGTCCATTCCATTTA-3'
Prdm6	5'-GTTGGCATGTGTTCTGCTTC-3'	5'-TTGGAGCAATAGGTGGTCTTC-3'
Rtn4rl2	5'-GGAGATCCCAAGAGGTATACTGA-3'	5'-TCCCAGAAACTGGCTTGATG-3'
Sema5a	5'-GGTGATGGGCTCAGAATCAA-3'	5'-CTTCCGACTCAGAAACTCTGTC-3'
Tmem176b	5'-CCACGTTCTCGGTTCTCTTT-3'	5'-GTCATAGTTCACAGCCAGGTAG-3'
Wwc2	5'-CTCTGGGCTGTCTTCTATCAC-3'	5'-GGGAGTTGTCATTCTTGCTAAA-3'
Zfhx3	5'-TGGATCAAGTGAGGGTCATCTA-3'	5'-TGTGAACCTGAAGCAGCTAATC-3'
Zfp608	5'-AGCCTCCAGATCAAAGGATAAC-3'	5'-TCTCTCTCTCTCTCTCTCTCTCT-3'
Tgfbr1	5'-CTCTGGAACTTAGCACCCTTT-3'	5'-GCACACTTTGGTACATTAACATCT-3'
Cd109	5'-CAGCTTGGGTGACAGTGATAC-3'	5'-CAGCTATTCTGACGTGCTTCT-3'
Ednrb	5'-GGTGGCGTCATTACCTCTTT-3'	5'-GGCGCGCAAACTTAACTTAC-3'
Efnb2-1	5'-CCAGCTATAAACTACATCACATGATA-3'	5'-TGAAGACAGTACAATAACTGGGT-3'
Efnb2-2	5'-AGATGGGCTGAACTGAATGAA-3'	5'-CCAGTTTGCTGCTGAATGTG-3'
Errfi1	5'-TTCTGTACCTGAGCTTACATC-3'	5'-GGTGAGAGATGACAGCATT-3'
Eya1	5'-ACTAGATGACAGTGGTTTCAACA-3'	5'-TCGAATATCCTGTTTGAGGAAGT-3'
Fgfr1	5'-GCTCCCAGTGCTGAAATCTA-3'	5'-GTAAACCTGCTTCTCCTGCT-3'
Nfib-1	5'-CTCCCTCTCTCGCTTTCTTTC-3'	5'-CACCGGTCTGATACCTTTAACA-3'
Nfib-2	5'-GGGCTAGGGCGCCGACTCTAG-3'	5'-CACGTGCACCGGGCGCCGGGG-3'
Shox2	5'-GGGAACTGGGAGTTATGAAGAG-3'	5'-GGTAAGGGTGCTGGGATTT-3'
Six1	5'-GTTGTGTGTGTGTGAGTGTATG-3'	5'-CTCTGACTGGGTCTACAAACTC-3'
Tbx18-1	5'-CAGAACCAGACTCGCAGAATAA-3'	5'-TCGTCAGTGTGTATACCTGTTTC-3'
Tbx18-2	5'-CTCACCTGAGGAATACCGAATG-3'	5'-TGGAGCATGTGTCCTTCTTAC-3'
Ovol2	5'-AGCTGCGGACAGAGACT-3'	5'-GAGAGTACCGAGCAACGC-3'
Ptn	5'-ACTGATTCTCAGACTCTCTCTCT-3'	5'-TTTAAATGCTGCTGTGAATGCT-3'
Sulf1-1	5'-ACCTGGCCCTACAAGAATTG-3'	5'-TGTAAGACTGAACCCTGAAACC-3'
Sulf1-2	5'-AGAGAGAAACACGGGCAGATA-3'	5'-AGGGAAAGCCTAAGGACACT-3'
Tgfbr1	5'-CGGCCAACACACAGTTTATC-3'	5'-AGCATGCAATGTTAATGCAA-3'
Trp63	5'-CAAGTTACTTCAGGACAGTATGC-3'	5'-CCCGTAGCCCAGTGATTTA-3'
Tff3	5'-TACGTTGGCCTGTCTCCAAG-3'	5'-CAGGGCACATTTGGGATACT-3'
Ins2	5'-GACCCACAAGTGGCACAAC-3'	5'-TCTACAATGCCACGCTTCTG-3'
Mup1	5'-TCTGTGACGTATGATGGATTCAA-3'	5'-TCTGGTTCTCGGCCATAGAG-3'
Spt1	5'-AACTTCTGGAACTGCTGATTCTG-3'	5'-GAGGCCTCATTAGCAGTGTTG-3'

Ttr	5'-GCTGTAGACGTGGCTGTAAA-3'	5'-CCACTCTGCTTTCTGACCTATC-3'
Amy2a	5'-ACTGGATTGGACCACCTAATAAC-3'	5'-GCCTCTGTTTCCTCTGCTAAA-3'
Afp	5'-CGTATTCCAACAGGAGGCTATG-3'	5'-GGCTCACACCAAAGAGTCAA-3'
Muc1	5'-GCTGGTGCTGGTCTGTATTT-3'	5'-CCACAGCTGGGTTGGTATAAG-3'

Table S2. OVOL2 immunoprecipitation experiments

Experiment	Run Number	Submission Date	Purpose	Details
1	824922	11/9/20	Negative Control-1	Empty-FLAG, No Treatment
1	824924	11/9/20	Negative Control-2	Empty-FLAG, Dnase I (20 unit)
1	824925	11/9/20	Negative Control-3	Empty-FLAG, Dnase I (100 unit)
1	824928	11/9/20	Co-immunoprecipitation-1	OVOL2-FLAG, No Treatment
1	824929	11/9/20	Co-immunoprecipitation-2	OVOL2-FLAG, Dnase I (20 unit)
1	824930	11/9/20	Co-immunoprecipitation-3	OVOL2-FLAG, Dnase I (100 unit)
2	833695	12/1/20	Negative Control-1	Empty-FLAG
2	833696	12/1/20	Co-immunoprecipitation-1	OVOL2-FLAG
2	833697	12/1/20	Negative Control-2	Empty-FLAG, Dnase I
2	833698	12/1/20	Co-immunoprecipitation-2	OVOL2-FLAG, Dnase I
3	889072	5/7/21	Negative Control-4	Empty-FLAG, No Treatment
3	889074	5/7/21	Negative Control-5	Empty-FLAG, No Treatment
3	889075	5/7/21	Negative Control-6	Empty-FLAG
3	889077	5/7/21	Negative Control-7	Empty-FLAG, Dnase I
3	889073	5/7/21	Co-immunoprecipitation-4	OVOL2-FLAG
3	889076	5/7/21	Co-immunoprecipitation-5	OVOL2-FLAG
3	889078	5/7/21	Co-immunoprecipitation-6	OVOL2-FLAG, Dnase I

REFERENCE

1. Baran-Gale, J. *et al.* Ageing compromises mouse thymus function and remodels epithelial cell differentiation. *Elife* **9** (2020).
