# Supplementary material for: OVOL2 sustains postnatal thymic epithelial cell identity
Source: Nat Commun. 2023 Nov 27;14:7786. doi: 10.1038/s41467-023-43456-z (PMC10682436; doi:10.1038/s41467-023-43456-z)
Supplement: Supplementary file 3 — Description of additional supplementary files [file 41467_2023_43456_MOESM3_ESM.pdf]

Description of additional supplementary files

Supplementary Data 1. Proteins identified in FLAG-OVOL2 and control immunoprecipitations by mass spectrometry.

Supplementary Data 2. Genes in accessible chromatin in Ovol2+/+ TECs.

Supplementary Data 3. Genes in accessible chromatin in Ovol2C120Y/C120Y TECs.

Supplementary Data 4. Gene Ontology pathway enrichment among genes accessible in Ovol2C120Y/C120Y TECs.

Supplementary Data 5. Gene Ontology pathway enrichment among genes accessible in Ovol2+/+ TECs.
