# Supplementary material for: OVOL2 sustains postnatal thymic epithelial cell identity
Source: Nat Commun. 2023 Nov 27;14:7786. doi: 10.1038/s41467-023-43456-z (PMC10682436; doi:10.1038/s41467-023-43456-z)
Supplement: Supplementary file 9 — Reporting Summary [file 41467_2023_43456_MOESM9_ESM.pdf]

nature portfolio

Corresponding author(s): Jin Huk Choi and Bruce Beutler

Last updated by author(s): Oct 30, 2023

Reporting Summary

Nature Portfolio wishes to improve the reproducibility of the work that we publish. This form provides structure for consistency and transparency in reporting. For further information on Nature Portfolio policies, see our Editorial Policies and the Editorial Policy Checklist.

Statistics

For all statistical analyses, confirm that the following items are present in the figure legend, table legend, main text, or Methods section.

n/a	Confirmed	
☐	☒	The exact sample size (*n*) for each experimental group/condition, given as a discrete number and unit of measurement
☐	☒	A statement on whether measurements were taken from distinct samples or whether the same sample was measured repeatedly
☐	☒	The statistical test(s) used AND whether they are one- or two-sided<br>*Only common tests should be described solely by name; describe more complex techniques in the Methods section.*
☒	☐	A description of all covariates tested
☐	☒	A description of any assumptions or corrections, such as tests of normality and adjustment for multiple comparisons
☐	☒	A full description of the statistical parameters including central tendency (e.g. means) or other basic estimates (e.g. regression coefficient) AND variation (e.g. standard deviation) or associated estimates of uncertainty (e.g. confidence intervals)
☐	☒	For null hypothesis testing, the test statistic (e.g. *F*, *t*, *r*) with confidence intervals, effect sizes, degrees of freedom and *P* value noted<br>*Give P values as exact values whenever suitable.*
☒	☐	For Bayesian analysis, information on the choice of priors and Markov chain Monte Carlo settings
☒	☐	For hierarchical and complex designs, identification of the appropriate level for tests and full reporting of outcomes
☒	☐	Estimates of effect sizes (e.g. Cohen's *d*, Pearson's *r*), indicating how they were calculated

Our web collection on statistics for biologists contains articles on many of the points above.

Software and code

Policy information about availability of computer code

Data collection	No software was used.
Data analysis	FlowJo 10 was used to analyze flow cytometry data. GraphPad Prism 9 was used to prepare graphs and to perform statistical analyses. Protein and peptide identification were done with MSFragger version 3.4 (https://fragpipe.nesvilab.org/). ATACseq data were analyzed using FastQC version 0.11.8 (for quality control of raw data), cutadapt version 2.5 (to remove adapter sequences), BWA (Burrows-Wheeler Aligner) version 0.7.17-r1188 (to align the reads to the reference genome), MACS2 (Model-based Analysis of ChIP-Seq) version 2.2.4 (for peak calling), TxDb.Mmusculus.UCSC.mm10.knownGene and the annotatePeak function in ChIPseeker version 1.36.0 (for peak annotation), and GREAT version 2.2.0 (to analyze biological functions of genes).

For manuscripts utilizing custom algorithms or software that are central to the research but not yet described in published literature, software must be made available to editors and reviewers. We strongly encourage code deposition in a community repository (e.g. GitHub). See the Nature Portfolio guidelines for submitting code & software for further information.

Data

Policy information about availability of data

All manuscripts must include a data availability statement. This statement should provide the following information, where applicable:

- Accession codes, unique identifiers, or web links for publicly available datasets
- A description of any restrictions on data availability
- For clinical datasets or third party data, please ensure that the statement adheres to our policy

The raw ATACseq data generated in this study have been deposited in Sequence Read Archive with the BioProject accession no. PRJNA914728 (https://www.ncbi.nlm.nih.gov/bioproject/PRJNA914728). The raw mass spectrometry data generated in this study have been deposited in ProteomeXchange with accession no. PXD039041 (https://proteomecentral.proteomexchange.org/cgi/GetDataset?ID=PXD039041). Source data are provided with this paper.

Human research participants

Policy information about studies involving human research participants and Sex and Gender in Research.

Reporting on sex and gender	No human participants were included in this study.
Population characteristics	NA
Recruitment	NA
Ethics oversight	NA

Note that full information on the approval of the study protocol must also be provided in the manuscript.

Field-specific reporting

Please select the one below that is the best fit for your research. If you are not sure, read the appropriate sections before making your selection.

☒ Life sciences ☐ Behavioural & social sciences ☐ Ecological, evolutionary & environmental sciences

For a reference copy of the document with all sections, see nature.com/documents/nr-reporting-summary-flat.pdf

Life sciences study design

All studies must disclose on these points even when the disclosure is negative.

Sample size	No pre-specified effect size was assumed, and in general three or more animals or replicates for each genotype or condition were used in experiments; this sample size was sufficient to demonstrate statistically significant differences in comparisons between two unpaired experimental groups by unpaired t-test or between multiple groups by ANOVA and post-hoc multiple comparisons test.
Data exclusions	No data were excluded from any analyses.
Replication	All experiments were repeated as mentioned in the text and and all attempts at replication were successful.<br>1. Data points represent individual mice.<br>2. In-vitro LSD1 or HDAC1 activity assay: Data points represent individual wells.<br>3. Real-time PCR: Data points represent individual sorting pools from 5 mice per pool.<br>4. ATACseq: Data points represent individual sorting pools from 20 mice per pool.<br>5. ChIP by CUT&RUN: Data points represent individual sorting pools from 5 mice per pool.
Randomization	The allocation of samples or animals to experimental groups was random.
Blinding	The investigator was blinded to genotypes during flow cytometry screening of ENU mutant mice and CRISPR mice (validation of original phenotypes). During other experiments, the investigator was not blinded to genotypes because the same investigator performed both genotyping and the experiments themselves.

Reporting for specific materials, systems and methods

We require information from authors about some types of materials, experimental systems and methods used in many studies. Here, indicate whether each material, system or method listed is relevant to your study. If you are not sure if a list item applies to your research, read the appropriate section before selecting a response.

Materials & experimental systems

n/a	Involved in the study
☐	☒ Antibodies
☐	☒ Eukaryotic cell lines
☒	☐ Palaeontology and archaeology
☐	☒ Animals and other organisms
☒	☐ Clinical data
☒	☐ Dual use research of concern

Methods

n/a	Involved in the study
☒	☐ ChIP-seq
☐	☒ Flow cytometry
☒	☐ MRI-based neuroimaging

Antibodies

Antibodies used

FACs antibodies (diluted 1:200):

1. Peripheral blood, spleen, kidney, lung, liver, intestine, and salivary gland: B220 (BD, clone RA3-6B2), CD19 (BD, clone 1D3), IgM (BD, clone R6-60.2), IgD (Biolegend, clone 11-26c.2a), CD3ε (BD, clone 145-2C11), CD4 (BD, clone RM4-5), CD8α (Biolegend, clone 53-6.7), CD11b (Biolegend, clone M1/70), CD11c (BD, clone HL3), F4/80 (Tonbo, clone BM8.1), CD44 (BD, clone 1M7), CD62L (Tonbo, clone MEL-14), CD5 (BD, clone 53-7.3), CD43 (BD, clone S7), NK 1.1 (Biolegend, clone OK136)), and 1:200 Fc block (Tonbo, clone 2.4G2)

2. B cells in bone marrow: IgM (BD, clone R6-60.2), IgD (Biolegend, clone 11-26c.2a), B220 (BD, clone RA3-6B2), CD43 (BD, clone S7), CD24 (Biolegend, clone M1/69), Ly-51 (BD, clone BP-1), CD19 (BD, clone 1D3), and 1:200 Fc block (Tonbo, clone 2.4G2)

3. Splenic B cells: CD4 (BD, clone RM4-5), IgM (BD, clone R6-60.2), IgD (Biolegend, clone 11-26c.2a), B220 (BD, clone RA3-6B2), CD23 (BD, clone B3B4), CD21/CD35 (Biolegend, clone 7E9), CD93 (BD, clone AA4.1), and 1:200 Fc block (Tonbo, clone 2.4G2)

4. Thymocytes: CD25 (Biolegend, clone 3C7), CD44 (BD, clone 1M7), CD4 (BD, clone RM4-5), CD8α (Biolegend, clone 53-6.7), CD69 (eBioscience, clone H1.2F3), CD5 (BD, clone 53-7.3), CD117 (c-kit) (Biolegend, clone 2B8), TCR-β (BD, clone H57-597), PD-1 (eBioscience, clone 29F.1A12), CD197 (CCR7) (Biolegend, clone 4B12), Helios (Biolegend, clone 22F6), and 1:200 Fc block (Tonbo, clone 2.4G2)

5. Hematopoietic stem and progenitors: Alexa Fluor 700 anti-mouse lineage cocktail [Biolegend, clones 17A2/RB6-8C5/RA3-6B2/Ter-119/M1/70), CD34 (eBioscience, clone RAM34), CD135 (Biolegend, clone A2F10), CD16/CD32 (eBioscience, clone 93), CD127 (BD, clone SB/199), CD135 (Biolegend, clone A2F10), Ly-6A/E (Sca-1) (Biolegend, clone D7), CD117 (c-kit) (Biolegend, clone 2B8), and 1:200 Fc block (Tonbo, clone 2.4G2)

6. Intracytoplasmic AIRE staining in mTECs and FoxP3+ regulatory T cell analysis in mesenteric lymph node and spleen: CD45 (eBioscience, clone 30-F11), Alexa Fluor 700 anti-mouse lineage cocktail (Biolegend, clones 17A2/RB6-8C5/RA3-6B2/Ter-119/M1/70), EpCAM (eBioscience, clone G8.8),I-A/I-E (Biolegend, clone M5/114.15.2), Aire (eBioscience, clone 5H12), CD3ε (BD, clone 145-2C11), CD4 (BD, clone RM4-5), CD8α (Biolegend, clone 53-6.7), CD25 (Biolegend, clone 3C7) and FoxP3 (BD, clone MF23)

6. CD45 congenic markers: CD45.1 (Biolgend, cloneA10), CD45.2 (Biolgend, clone 104), and 1:200 Fc block (Tonbo, clone 2.4G2)

7. Thymic stromal cells including TECs, mesenchymal cells and endothelial cells: CD45 (eBioscience, clone 30-F11), Alexa Fluor 700 anti-mouse lineage cocktail (Biolegend, clones 17A2/RB6-8C5/RA3-6B2/Ter-119/M1/70), EpCAM (eBioscience, clone G8.8), CD31 (Biolegend, clone 390), CD140a (PDGFR-α) (Biolegend, clone APA5), CD140b (PDGFR-β) (Biolegend, clone APB5), CD80 (Biolegend, clone 16-10A1), CD86 (Biolegend, clone GL-1), podoplanin/gp38 (Biolegend, clone 8.1.1), H-2Kb (eBioscience, clone AF6-88.5.5.3), Ly51 (BD Pharmingen, clone 6C3), UEA-1 (Vector Laboratories, clone FL-1061), I-A/I-E (Biolegend, clone M5/114.15.2), and 1:200 Fc block (Tonbo, clone 2.4G2)

Western blot antibodies:

1. Primary antibodies (diluted 1:1000): anti-HA (Cell Signaling Technology, clone C29F4), anti-Flag (Sigma Aldrich, clone M2), anti-LSD1 (CST, clone C69G12), anti-HDAC1 (CST, clone D5C6U), anti-HDAC2 (CST, clone D6S5P), anti-RCOR1 (Thermo Fisher Scientific, AF6047-SP), anti-α-Tubulin (CST, clone DM1A), anti-H3 (Abcam, ab1791), anti-H3K4me2 (Thermo Fisher Scientific, 39141), anti-GAPDH (CST, clone D16H11), anti-OVOL2 (Santa Cruz, clone E-9), and anti-β-Actin (CST, clone 8H10D10)

2. Secondary antibodies (diluted 1:4000): goat anti-mouse IgG-HRP (Southern Biotech), goat anti-rabbit IgG-HRP (Thermo Fisher Scientific), or donkey anti-sheep IgG-HRP (Santa Cruz)

Immunohistochemistry antibodies (diluted 1:50):
1. Thymus sections: cytokeratin 8 (CK8) (ABclonal, clone A1024), cytokeratin 5 (CK5) (ABclonal, clone A2662)

CUT&RUN antibodies (diluted 1:50):
1. Di-Methyl-Histone H3 (Lys4) antibody (Active Motif, AB_2614985)

Validation

All antibodies used in this study were previously validated for the specific application by the companies from which we purchased them. Validation information including relevant citations can be found on the product webpage at the manufacturer's website:

BD (https://www.bdbiosciences.com/en-us)
Biolegend (https://www.biolegend.com/)
Tonbo Biosciences (https://cytekbio.com/collections/tonbo-reagents)
eBioscience (https://www.thermofisher.com/us/en/home/life-science/antibodies/ebioscience.html)
Vector Laboratories (https://vectorlabs.com/)
Cell Signaling Technology (CST) (https://www.cellsignal.com/)
Sigma-Aldrich (https://www.sigmaaldrich.com/US/en)
Thermo Fisher Scientific (https://www.thermofisher.com/us/en/home.html)
Santa Cruz Biotechnology (https://www.scbt.com/home)
Southern Biotech (https://www.southernbiotech.com/)
ABclonal (https://abclonal.com/)
Active Motif (https://www.activemotif.com/)

Eukaryotic cell lines

Policy information about cell lines and Sex and Gender in Research

Cell line source(s)	Human HEK293T cells (female) and mouse EL4 cells (sex unspecified) were purchased from ATCC.
Authentication	HEK293T cells were authenticated by STR profiling performed by ATCC. EL4 cells were not authenticated.
Mycoplasma contamination	The cell lines were not tested for mycoplasma contamination.
Commonly misidentified lines (See ICLAC register)	Commonly misidentified cell lines were not used in this study.

Animals and other research organisms

Policy information about studies involving animals; ARRIVE guidelines recommended for reporting animal research, and Sex and Gender in Research

Laboratory animals	The following mouse strains (Mus musculus) were used in this study: C57BL/6J, C57BL/6.SJL (B6.SJL-Ptprc<a> Pepc<b>/BoyJ), Rag2-/- (B6.Cg-Rag2<tm1.1Cgn>/J), Foxn1-Cre [B6(Cg)-Foxn1<tm3(cre)Nrm>/J], and Ai9 [B6.Cg-Gt(ROSA)26Sor<tm9(CAG-tdTomato)Hze>/J]. The Ovol2<C120Y> mutation, loxP flanked Ovol2, 3xFlag tagged Ovol2, and Grhl2<clayton> mutation were generated in mice on a C57BL/6J background. Mice were 6 to 20 weeks of age when used in experiments (indicated in Methods or legends). Conventionally reared mice were housed in specific pathogen–free conditions, 12 h light/12 h dark cycle, 20-26°C ambient temperature, and 30-70% humidity at the University of Texas Southwestern Medical Center.
Wild animals	This study did not involve wild animals.
Reporting on sex	Sex was not considered in study design and males and females were analyzed together.
Field-collected samples	This study did not involve samples collected from the field.
Ethics oversight	All protocols were approved by the UT Southwestern Medical Center Institutional Animal Care and Use Committee (IACUC).

Note that full information on the approval of the study protocol must also be provided in the manuscript.

Flow Cytometry

Plots

Confirm that:

☒ The axis labels state the marker and fluorochrome used (e.g. CD4-FITC).

☒ The axis scales are clearly visible. Include numbers along axes only for bottom left plot of group (a 'group' is an analysis of identical markers).

☒ All plots are contour plots with outliers or pseudocolor plots.

☒ A numerical value for number of cells or percentage (with statistics) is provided.

Methodology

Sample preparation	1. Peripheral blood immune cells: Blood was collected from mice >6 weeks old by cheek bleeding. Red blood cells (RBCs) were lysed with hypotonic buffer (eBioscience). Samples were washed with FACS staining buffer (PBS with 1% (w/v) BSA) one time and then centrifuged at 500 × g for 5 minutes. The RBC-depleted samples were stained for 1 hour at 4°C, in 100 µl of appropriately diluted fluorescence-conjugated antibodies and 1:200 Fc block (Tonbo, clone 2.4G2).<br><br>2. For the lymphocytic infiltration analysis, kidney, lung, liver, intestine, and salivary gland were digested in PBS containing 0.5 U/mL Liberase TM (Sigma-Aldrich) and 0.02% (w/v) DNase I (Roche) to produce single cell suspensions before antibody staining.

3. B, T, hematopoietic stem and progenitor cells: Bone marrow cells, thymocytes, and splenocytes were isolated, plunged, and passed a 40 μm cell strainer. RBC lysis buffer was added to remove RBCs. Cells were stained at 1:200 dilution in the presence of anti-mouse CD16/32 antibody (Tonbo, clone 2.4G2) and different antibody cocktails.

4. For the intracytoplasmic AIRE staining in mTECs and FoxP3+ regulatory T cell analysis in mesenteric lymph node and spleen, after a first staining with antibodies for surface markers, cells were collected, fixed, permeabilized with Cytofix/Cytoperm (BD Bioscience), and stained with intracellular markers.

5. Thymic stromal cells: Thymic tissues were cut into small pieces by forceps and placed into a 15-mL tube containing 2mL of RPMI-1640 (Gibco) + 1% fetal calf serum (FCS). After pipetting and settling for 2 min, the supernatant was discarded. This was repeated several times. RPMI-1640 + 1% FCS containing 0.5 U/mL Liberase TM (Sigma-Aldrich) and 0.02% (w/v) DNase I (Roche) were added to remaining thymic fragments and incubated at 37°C for 12 min. After settling for 2 min, the supernatant was collected into a 15- mL tube and suspended with PBS (minus Ca2+ and Mg2+) + 1% FCS + 5 mM EDTA. This step was repeated twice. After washing cells, they were passed through mesh. Single cell suspensions were stained with antibodies.

Instrument

BD LSRFortessa (BD Biosciences)

Software

FlowJo 10 was used to analyze FACS data.

Cell population abundance

The post-sort abundance of relevant cell populations in the peripheral blood and tissues of mice is well established. We found that all tested populations were sufficiently abundant to gate and quantitate, and they were within a reasonable range. The purity of cell populations was determined using commercially available monoclonal antibodies against the specific surface markers on each cell type.

Gating strategy

For all experiments, cells were initially gated based on their size in a FSC-A vs SSC-A scatter plot. Aggregates were then excluded based on FSC-H vs FSC-A scatter plots. Gating was as follows:

1. Peripheral blood, spleen, kidney, lung, liver, intestine, and salivary gland: B cells (B220+); CD3+ T cells (CD3ε+); CD4+ T cells (CD4+); CD8+ T cells (CD8α+); NK cells (NK-1.1+ CD3ε-); neutrophils (CD11b+ F4/80-); macrophages (CD11b+ F4/80+); B1 cells (B220low CD19+); dendritic cells (CD11c+); naive T cells (CD44low CD62L+); central memory T cells (CD44high CD62L+); effector memory T cells (CD44high CD62L-); B1a cells (CD5+ CD43+); B1b cells (CD5- CD43+); B2 cells (CD5- CD43-), NK T cells (NK-1.1+ CD3ε+).

2. B cells in bone marrow: pre-proB (B220low BP-1- CD24low); pro-B (B220low BP-1- CD24+); pre-B (B220low BP-1+ CD24+); immature B (B220+ CD43+ CD24+ IgM+ IgD-); transitional B (B220+ CD43+ CD24+ IgMhigh IgDlow); mature recirculating B (B220+ CD43+ CD24+ IgD+ IgM+).

3. Splenic B cells: T1 (B220+ CD93+ IgMhigh CD23−); T2 (B220+ CD93+ IgMhigh CD23+); T3 (B220+ CD93+ IgMlow CD23+); follicular B (FOB) (B220+ CD93− IgM+ CD21+ CD23high); marginal zone precursor (MZP) (B220+ CD93− IgM+ CD21+ CD23high); marginal zone B (MZB) (B220+ CD93− IgM+ CD21+ CD23low).

4. Thymocytes: ETP (CD44+c-Kit+CD25low); DP (CD4+CD8+); SP4 (CD4+CD8-); SP8 (CD4-CD8+); DN1 (CD4-CD8-CD25-CD44+); DN2 (CD4-CD8-CD25+CD44+); DN3 (CD4-CD8-CD25-CD44-); DN4 (CD4-CD8-CD25-CD44-); positive selection: population A (CD69-TCRlow), population B (CD69+TCRint), population C (CD69+TCRhigh), population D (CD69-TCRhigh), population i (TCRβloCD5lo), population ii (TCRβloCD5int), population iii (TCRβintCD5hi), population iv (TCRβhiCD5hi); negative selection: see Supplementary Fig. 10.

5. Treg cells: CD4+Foxp3+CD25int

6. Hematopoietic stem and progenitors: Single cells were analyzed on the basis of being lineage cocktail (Lin: B220, CD3, CD11b, Ly-6G/6C, and Ter-119 antibodies) negative or positive. Lin[-] cells were further gated as follows: LK (Lineage- c-Kit+ Sca-1-); LSK (Lineage- c-Kit+ Sca-1+); CLP (Lineage- c-Kitlow Sca-1low IL-7Rα+); MPP (Lineage- c-Kit+ Sca-1+ Flk2+ CD34+); LT-HSC (Lineage- c-Kit+ Sca-1+ Flk2- CD34-); ST-HSC (Lineage- c-Kit+ Sca-1+ Flk2- CD34+); GMP (Lineage- c-Kit+ Sca-1- CD16/32+ CD34+); MEP (Lineage- c-Kit+ Sca-1- CD16/32- CD34-); CMP (Lineage- c-Kit+ Sca-1- CD16/32- CD34+).

7. Thymic epithelial cells (TECs): TECs (CD45- EpCAM+); mTECs (CD45- EpCAM+ UEA-1+ Ly51-); cTECs (CD45- EpCAM+ UEA-1- Ly51+)

8. Non-TEC thymic stromal cells: endothelial cells (EpCAM-CD31+); mesenchymal cells (EpCAM-CD31-PDGFR-α/β+); fibroblasts (EpCAM-CD31-PDGFR-α/β+Ly51intgp38+)

☒ Tick this box to confirm that a figure exemplifying the gating strategy is provided in the Supplementary Information.
